# Out of Line or Altered States? Neural Progenitors as a Target in a Polygenic Neurodevelopmental Disorder

**DOI:** 10.1159/000530898

**Published:** 2023-05-10

**Authors:** Shah Rukh, Daniel W. Meechan, Thomas M. Maynard, Anthony-Samuel LaMantia

**Affiliations:** a Fralin Biomedical Research Institute, Virginia Tech Carilion School of Medicine, Roanoke, VA, USA;; b Department of Biological Sciences, Virginia Tech, Blacksburg, VA, USA

**Keywords:** 22q11 deletion syndrome, Animal model, Cell lineage, Cerebral cortex, Neural development, Neurogenesis

## Abstract

The genesis of a mature complement of neurons is thought to require, at least in part, precursor cell lineages in which neural progenitors have distinct identities recognized by exclusive expression of one or a few molecular markers. Nevertheless, limited progenitor types distinguished by specific markers and lineal progression through such subclasses cannot easily yield the magnitude of neuronal diversity in most regions of the nervous system. The late Verne Caviness, to whom this edition of *Developmental Neuroscience* is dedicated, recognized this mismatch. In his pioneering work on the histogenesis of the cerebral cortex, he acknowledged the additional flexibility required to generate multiple classes of cortical projection and interneurons. This flexibility may be accomplished by establishing cell states in which levels rather than binary expression or repression of individual genes vary across each progenitor’s shared transcriptome. Such states may reflect local, stochastic signaling via soluble factors or coincidence of cell surface ligand/receptor pairs in subsets of neighboring progenitors. This probabilistic, rather than determined, signaling could modify transcription levels via multiple pathways within an apparently uniform population of progenitors. Progenitor states, therefore, rather than lineal relationships between types may underlie the generation of neuronal diversity in most regions of the nervous system. Moreover, mechanisms that influence variation required for flexible progenitor states may be targets for pathological changes in a broad range of neurodevelopmental disorders, especially those with polygenic origins.

## Introduction

Neuronal stem and progenitor cells have been suggested as prime targets for pathogenesis in a broad range of clinically diagnosed neurodevelopmental disorders (NDDs) including intellectual disability (ID), autistic spectrum disorder (ASD), attention deficit/hyperactivity disorder (ADHD), and schizophrenia (Scz). This suggestion was presaged by the pioneering work of Verne Caviness and his colleagues, beginning in the 1970s. Verne’s early work defined neural progenitor dynamics in the developing cerebral cortex, cerebellum, and hippocampus. It also established the vulnerability of these progenitors to single gene mutations that compromise neurogenesis in “neurological mutant” mice (reviewed by [1–4]). The subsequent arguments for NDD-mediated neural progenitor pathology were based primarily upon changes in numbers of neuronal progeny in the apparent absence of extensive cell death, especially in the neocortex of genetic NDD animal models including those based upon single genes mutated in humans with a broad range of NDD diagnoses [5–7]. In humans with clinically or genetically defined NDDs, disrupted neurogenesis in the cortex and other sites has been inferred from postmortem tissue analyses or gray matter thickness measurements in structural imaging studies [8–10]. Parallel analyses of single gene mutations as well as polygenic disruptions, especially copy number variants (CNVs) associated with NDD risk in genomically accurate animal models, has identified disrupted neural progenitor proliferation and neurogenic capacity as key contributors to pathogenesis underlying circuit dysfunction and behavioral deficits [11–13]. In these instances, neural progenitor proliferative characteristics and subsequent neurogenesis are altered; however, the mechanisms underlying many of these changes remain uncertain.

Inspired by Verne’s work on neurological mutant mice and the use of genetic approaches to better understand neural progenitor dynamics and NDD pathology it inspired, we have analyzed the consequences of polygenic, dosage-based CNV disruption for multiple classes of neural precursors in mouse models of 22q11.2 deletion syndrome (22q11DS). 22q11DS is one of the most frequent CNV syndromes, and it is associated with substantially elevated risk for multiple clinically defined NDDs including ID, ASD, ADHD, and Scz. Based upon our observations in the genomically accurate *LgDel* 22q11DS mouse model (heterozygous deletion on mouse chromosome 16 of 28/32 contiguous orthologues of human 22q11.2 deleted genes [3, 14–16]), as well as consequences of mutations of single genes within the region, we suggest that progenitors within broadly defined molecular classes acquire quantitative “states” that regulate their neurogenic capacity instead of progressing through precisely defined lineages. Such state-dependent mechanisms provide flexibility to generate appropriate numbers of neurons with diverse but malleable differentiation programs for optimal circuit organization and function. These probabilistic, rather than deterministic, progenitor states may be more susceptible to pathology due to CNVs or unique combinations of single gene variants thought to underlie polygenic origins of a large proportion of NDDs [8, 17].

## Neural Progenitors as a Target for NDD Pathogenesis

Long before current classification of clinically diagnosed behavioral diseases – including ID, ADHD, ASD, and Scz – as NDDs, disrupted development was a suspect for pathogenesis in these disorders. Some NDD disruptions, particularly those resulting in ID, were associated with perinatal hypoxia/ischemia or other birth-related complications [18, 19] that result in NDD-related behavioral deficits. Nevertheless, evidence for altered cortical neurogenesis during initial brain development in ASD, Scz, and many other clinically diagnosed NDDs remains indirect – rigorous analysis of neurogenesis in human fetal material, where risk for these diseases in the absence of diagnostic behavioral deficits can (at best) be inferred is difficult if not impossible. Altered neurogenesis has been suggested as a potential explanation for differences in cortical size, functional connectivity, neuronal density, and transcriptome divergence associated with ASD as well as ADHD [8, 20, 21] or Scz [22–24]. Many other mechanistic explanations, however, could account for these changes in the mature brains of individuals with ASD, ADHD, or Scz. Ventricular heterotopias and cortical dysplasia in individuals with ASD may reflect disrupted cortical neurogenesis ([25, 26], reviewed by [27]). This interpretation is supported by recent cellular analyses of the roles of multiple ASD risk genes in neurogenesis and neuronal migration [28, 29]. Observations using induced pluripotent stem cells (IPSCs) derived from individuals with Scz suggest that IPSC-derived precursors have divergent capacities for proliferation and self-organization into cortex-like aggregates (reviewed by [30]). Despite these observations, establishing a causal relationship between altered cortical progenitor proliferation and key behavioral pathologies in NDDs like Scz and ASD remains challenging. Nevertheless, cortical neurogenesis remains a compelling, if speculative, candidate for pathogenesis in common clinical NDDS like ASD and Scz because of its central role in producing projection neurons (PNs) from which association cortico-cortical circuits – presumed prime targets of NDD pathology [31, 32] – are built.

The earliest genetic and cell biological models for pathological neurogenesis in NDDs were based upon observations, many made by Verne and his collaborators (particularly Pasko Rakic), in multiple strains of “neurological mutant” mice [2]. Monogenic mutations in these mice were identified initially by mode of inheritance and distinct behavioral phenotypes (reeling, lurching, staggering, weaving) without knowledge of a specific gene or protein product target. Many of these mutations were shown to selectively disrupt differentiation of neurons and their arrangement into orderly cortices in the cerebellum, hippocampus, and cerebrum [2, 33–35]. The likely relevance of these early studies in mutant mice to understanding cortical malformations in humans, as well as the impact of altered genetic architecture on connections and function of neural circuits, were acknowledged by Verne and Pasko throughout their now classic 1978 review “Mechanisms of Cortical Development: A View from Mutations in Mice” [2]. Nevertheless, they recognized at the time (45 years ago!) that few single gene mutations had been associated with altered cortical development in humans and thus wisely refrained from additional speculation of how such disruptions result in what are now recognized as NDDs.

Seventeen years later, in 1995 [1], when Verne and Pasko revisited a central “neurological mutant” *reeler*, described at length in their 1978 review, they were more confident of the relevance of the genetic approach for understanding human cortical malformations and their consequences. Summarizing the significance of the identification of the *reeler* gene in mice and humans in this context:

“Molecular and cell biological explorations of the role of the reeler gene in normal cortical histogenesis now take their place in a widening strategy of analysis of normal and abnormal histogenesis. Mutations associated with consistent patterns of cortical malformation in man will figure preeminently in this strategy” [1].

Thus, observations in *reeler* and other neurological mutant mice anticipated, and in some ways inspired, the confluence of human genetics, human in vivo imaging, and clinical characterization of a broad range of primarily monogenic behavioral disorders as well as parallel cellular and molecular neuroscience in animal models. Together, these observations eventually confirmed that NDDs can indeed be as disorders of neurogenesis, neuronal migration, and/or subsequent cortical circuit development (reviewed by [17, 32, 36]).

In the intervening years, remarkable progress has been made in establishing the key role of cortical histogenesis in NDD pathology based in large measure upon identification of multiple human monogenic disorders that result in microcephaly (reviewed by [37, 38]) as well as subsequent defects in migration of postmitotic cortical neuroblasts (reviewed by [39, 40]). Some of these mutations target genes that control brain size by modulating cell proliferation and division [41]. In addition, many human mutations, including autosomal recessive mutations in the human *RELN* gene [42], compromise the next step in cortical histogenesis, migration of postmitotic cortical neurons (reviewed by [43–45]). *RELN* in humans (and mice [46]) encodes an extracellular matrix protein that acts as a ligand to regulate neuronal migration and placement. RELN receptors and signaling mediators include cell surface proteins VLDLR, ApoER2, and the β1 integrin as well as additional interacting proteins like Dab1. RELN binding and subsequent activation of these proteins influences cell adhesion and motility [47–49]. Distinctions between microcephaly and migration disorders suggest a boundary between the two processes: there may be “neurogenesis genes” that control cell cycle and cytokinesis and “migration genes” that control adhesion, signaling, cytoskeletal integrity, and motility for postmitotic neurons. Verne and his colleagues, however, recognized that neurogenesis versus migration most likely did not define an absolute boundary of molecular functions in the developing cortex [50, 51]. Based upon a series of studies of cortical progenitor proliferative dynamics and their modulation by cell cycle regulators and downstream transcription factors [52, 53] as well as cell-cell interactions mediated by Notch signaling [54, 55], Verne et al. [1] suggested that the control of cortical neurogenesis via pathways not directly engaged in cell cycle regulation, cytokinesis, or even cell adhesion and migration can influence cortical neuron laminar identity, regional specificity, and circuit differentiation.

**Fig. 1.**
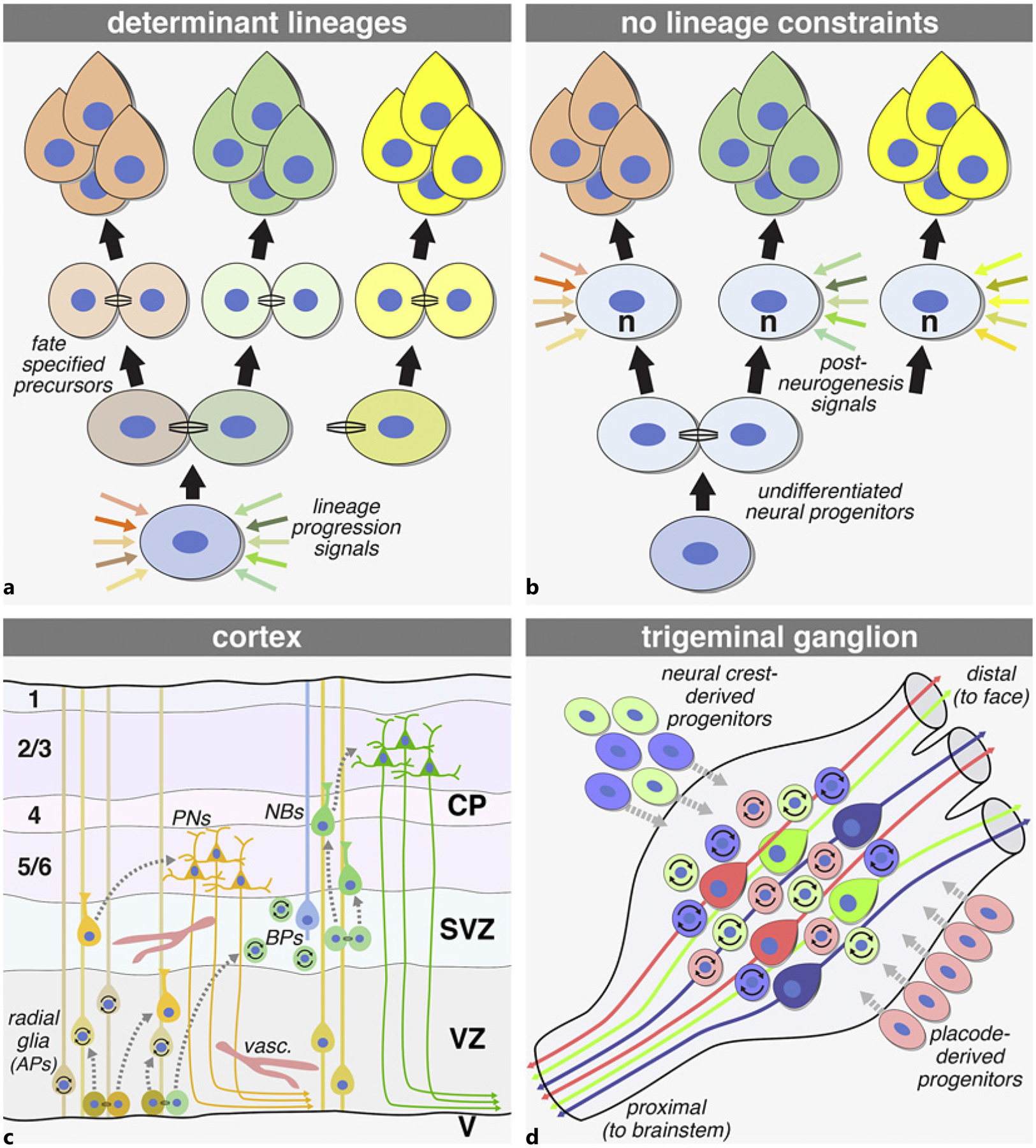
Neural progenitor specification and its relationship with CNS and PNS neuronal diversity. **a** Neuronal diversity may depend upon progenitor identities (distinct colors in dividing cells) that determine differentiation capacity of postmitotic progeny (related colors, top). Extrinsic signals may establish or reinforce discrete progenitor identities (multicolored arrows) during lineage progression. **b** Neuronal diversity may be independent of lineage, relying primarily upon combinations of extrinsic signals (multicolor arrows) that act on postmitotic neuroblasts to establish distinct identities. **c** Current views of lineage progression in the cerebral cortex. Radial glia (apical progenitors [AP]) in the ventricular zone ([VZ], adjacent to the cerebral ventricles [V]) primarily generate projection neurons (PNs) in layers 5/6 early in cortical histogenesis. They also act as guides for postmitotic neuroblasts (NBs) as they migrate radially to their laminar positions. At later stages, radial glia generate basal progenitors (BPs) which diversify in the subventricular zone (SVZ) to include basal radial glia (blue cell with process). BPs generate primarily layer 2/3 PNs. **d** Trigeminal ganglion progenitor diversity and presumed lineages. Two distinct neural crest progenitor types: one from Wnt1-expressing neuroepithelial neural tube progenitors (green) and another from non-Wnt1 neural tube progenitors (blue), migrate and coalesce with trigeminal cranial placode-derived progenitors (red). These populations, with distinct derivations, give rise, respectively, to mechanosensory (red) and nociceptive neurons (green and blue) axons extending into the distal and proximal branches of the trigeminal nerve (**c** after [60, 61]; **d** based upon data from [16, 62]).

It remains uncertain whether gene networks and local signaling engaged by these additional contributors can establish discretely defined progenitor cell types identified by singular molecular markers that are specified to generate distinct neuron classes in the cerebral cortex or indeed anywhere in the CNS or PNS (Fig. 1a). It is also possible that dynamic, statistically variable cell states, established by transient quantitative modulation of expression independent of singular molecular markers (reviewed by [56–59]; Fig. 1b), bias postmitotic neuroblasts toward potential fates. Such variable, dynamic, progenitor cell states would establish a far greater range of transcriptional activation and thus provide more flexible outcomes than those possible within a rigidly prescribed lineage defined by binary expression of single genes. These progenitor states may reflect differing probabilities of local signaling via soluble factors as well as cell surface ligands and receptors. They may also rely upon epigenetic regulation of transcription including DNA methylation and histone modifications that modify chromatin conformation and levels or probability of gene expression. In each instance, progenitor states would change quantitatively without altering binary expression distinctions that identify currently established progenitor types in prescribed lineages. The contrasting possibilities of progenitor types or states are particularly relevant for analyzing how cortical histogenesis – a term used frequently by Verne – is targeted by genetic or environmental mechanisms that result in clinically/behaviorally defined NDDs like ASD, ADHD, and Scz.

Recent analyses of disrupted neurogenesis associated with heterozygous deletion of the genes associated with 22q11DS, a polygenic CNV NDD that elevates risk for behavioral deficits parallel to those in ID, ADHD, ASD, and Scz, address fundamental questions raised by Verne and colleagues regarding dynamic regulation of neurogenesis and its impact on establishing optimal versus pathological neuron identities. Analyses in 22q11DS mouse models suggest that broad progenitor classes may be targeted by diminished dosage of 22q11 genes; however, these results also suggest that probabilities of divergent or disrupted proliferation and neurogenesis for individual progenitors within broad classes defined by singular molecular markers vary substantially. We argue that CNS and PNS neural precursor dysregulation in 22q11DS mouse models reflects mechanisms that destabilize cell states rather than disrupt specific progenitor types. Accordingly, variable quantitative, probabilistic cell states underlie divergent neurogenesis and circuit differentiation in NDDs, especially those with polygenic origins.

## Studying Neurogenesis: Locations, Signals, Lineages, and States

The fundamental challenge to understanding neurogenesis in any part of the nervous system is relating remarkable neuronal (and glial) diversity – defined cytologically, molecularly, and anatomically as well as by connections and physiological function – to a far more limited number of known progenitor types defined by expression of single molecular markers. It seems likely that the two extremes – rigidly determined lineages and postmitotic tabule rasa (see Fig. 1a, b) – are integrated so that progenitors within broad classes retain flexibility to produce postmitotic neuroblasts with biased fates that can subsequently acquire additional molecular distinctions to achieve maximal diversity. At least two cell biological mechanisms could mediate less rigidly determined changes of cell states within molecular marker-defined populations of CNS or PNS neural progenitors: (1) soluble extracellular signals available widely to responsive target precursors and external cell surface ligand/receptor partners whose signaling relies upon cell-cell contact between immediately adjacent precursors (Fig. 1b). These mechanisms, because of their flexibility and the large number of molecular mediators they engage, may also be targets of multigenic disruptions that underlie NDDs.

Soluble signals, including regulators of fundamental developmental mechanisms like induction and patterning, including Shh, Fgfs, BMPs, Wnts, and retinoic acid [63–69], cytokines [70, 71], morphogenetic signals including EGF [72] and VEGF [73], and even neurotransmitters [74, 75], are known to regulate neural progenitor proliferation and modes of division, both in vitro and in vivo. In the embryonic periphery, such signals released from nascent vasculature or by secretion from adjacent cells and tissues into the extracellular space [76–78] likely reach neural progenitors that generate peripheral sensory and autonomic ganglia and the enteric plexus. In addition, signals from amniotic fluid may access neural progenitors in neurogenic placodes that generate olfactory receptor neurons as well as those that contribute to the nascent sensory ganglia of the developing head [79]. Signals in amniotic fluid could also influence the enteric nervous system via fetal fluid ingestion [80]. In the central nervous system, these signals can be provided via the cerebrospinal fluid [80, 81] as well as local secretion by subsets of progenitors or differentiating neurons: e.g., Shh by Purkinje cells for granule cell precursors [82, 83], retinoic acid by subsets of adjacent spinal cord neurons [84, 85], multiple signals from differentiating glia or immune cells [86], circulating signals from developing brain vasculature [87, 88].

Neural progenitors also respond to contact-mediated signaling facilitated by direct interactions with neighboring cells. Such direct receptor-ligand interactions require membrane-to-membrane contact between progenitors that either signal or respond to their immediate neighbors. Thus, the position of each individual progenitor, its complement of cell surface receptors and ligands, and those of immediately adjacent cells – other progenitors, newly generated neurons and glia, vascular cells, or peripheral target tissues – is critical for establishing its proliferative and neurogenic capacity. Signals from immediate neighbors likely provide an additional, essential regulation of proliferation, modes of division, and initial differentiation capacity. These signals include ligands and receptors of the Notch/Delta pathway [54, 55], the Eph/Ephrin pathway [89, 90], and the cadherin/catenin pathway [91]. In each instance, cell positions and the direction of interaction based upon differential signal transmission or transduction capacity of receptor/ligand pairs may result in individual responses that distinguish immediately adjacent, otherwise apparently equivalent, neural progenitors. For Notch/Delta signaling, the consequences of direct local interaction include maintenance of progenitor state versus progression toward differentiation. Progenitor state dynamics via Notch signaling-mediated reflect transcriptional responses that influence proliferation as well as mode and plane of cell division: symmetric/asymmetric, self-renewing/neurogenic, and in the developing cortex, perpendicular or parallel to the ventricular surface [55, 92–94]. The influence of other direct cell-cell contact signaling pathways is less well characterized, but in each case, ligand/receptor interactions that depend upon direct contact between adjacent cells elicit intracellular signaling changes that influence progenitor proliferation and differentiation.

Soluble and cell-contact-dependent signals most likely diversify progenitor proliferation and the fates of their progeny beyond that inferred from classifications using a single or limited combination of molecular markers [60, 61, 95]. Nevertheless, the identification of molecular marker-defined precursors (Fig. 1c, d) throughout the developing and mature CNS and PNS [96–103] has led to inferences of lineages considered parallel to those defined for hematopoietic differentiation. Indeed, over the last three decades, the term “neuropoiesis” has been coined to emphasize general as well as specific similarities between hematopoietic lineages and apparent progression through molecularly labeled neural progenitor subclasses to distinct postmitotic neuronal progeny [104–107]. These comparisons have identified broadly shared characteristics of all stem cells – e.g., transcriptional regulation of proliferative states, the cell cycle, or cellular mechanisms for cytokinesis – that can be applied to neurogenic progenitors. There are, however, significant limitations. Hematopoiesis and its parallel, neuropoiesis, in simplest forms generate a limited number of cell types from a parallel set of molecularly distinct progenitors [108] and thus cannot easily accommodate cellular diversity in the CNS or PNS. Strictly applied, neuropoiesis anticipates linear progression through progenitor types that generate committed postmitotic neuroblasts that differentiate into a singular identity. This sort of linear progression, however, especially in the mammalian CNS and PNS, may not facilitate adjustments of identity and function necessary for flexible neural circuit differentiation including neuron/target interactions and activity-dependent plasticity that occur long after neurons become postmitotic.

A new synthesis is needed to integrate the contribution of linear progression through neural progenitor classes defined by one to a few molecular markers and the transcriptional flexibility needed as progenitors generate highly diverse and adaptable neurons that comprise complex and dynamic circuits of the mammalian brain. Verne recognized that this synthesis was necessary to fully explain typical neurogenesis in the cortex as well as its likely atypical execution in multiple NDDs. The substantial progress in identifying rare monogenic human diseases that compromise neural progenitors broadly and cerebral cortex precursors specifically or selectively [17, 44, 109–112] has provided essential insight into singular vulnerabilities of neural progenitors to genetic disruption. There is also, however, a need to understand how more subtle changes in neurogenesis arise in the context of the apparently probabilistic, polygenic disruptions [113, 114] associated with clinically defined NDDs including (but not limited to) ADHD [115, 116], ASD [8, 117], and Scz [118, 119]. Our analyses of CNS and PNS neurogenesis (Fig. 1c, d) in the “model” CNV syndrome, 22q11DS [4, 14, 75] using genomically valid mouse models [75] provide insight into how polygenic disruption of neural progenitors might contribute to circuit pathology and behavioral dysfunction [3, 15]. We argue that these polygenic disruptions may target progenitor “states” that reflect highly variable quantitative regulation of gene expression, cell-cell interactions, and modes of cell division.

## 22q11 Genes and Neural Precursor Diversity

**Fig. 2.**
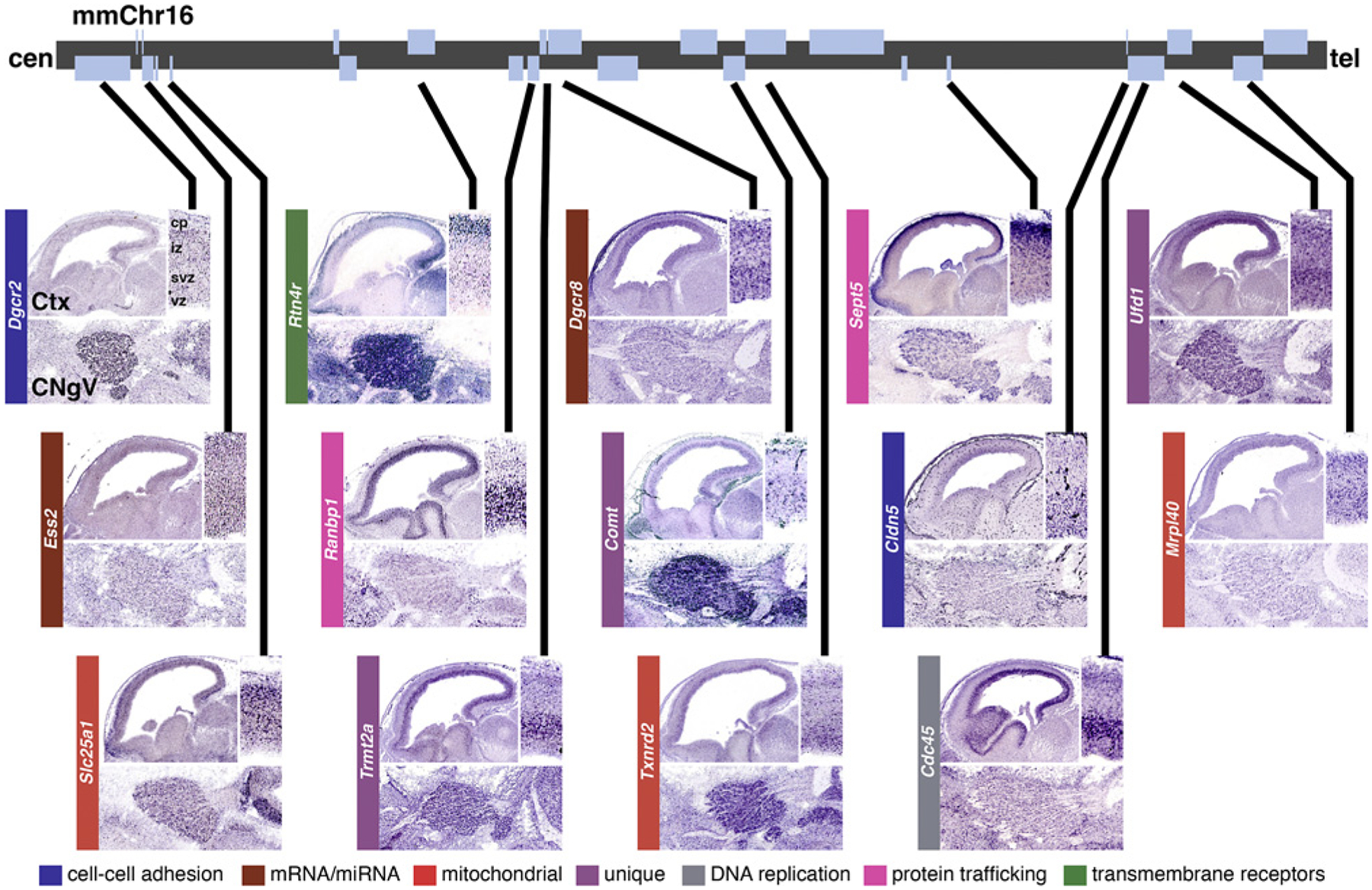
Multiple contiguous mouse orthologues on mouse chromosome 16 of genes that define the region of human chromosome 22 deleted in 22q11.2 deletion syndrome are selectively expressed in the VZ/SVZ and trigeminal ganglion during neurogenesis. Top: a schematic of the region of mouse chromosome 16 where 28 colinear orthologues of the genes from the minimal critical deleted region associated with 22q11DS are located. Gray bars indicate the loci of protein-coding genes. Several 22q11 genes are expressed selectively in the developing cortical mantle (top, each panel) as well as the trigeminal ganglion (CNgV; bottom, each panel) during cortical histogenesis or gangliogenesis. Lines extend from protein-coding loci on the chromosomal map to identify images of cortical and CNgV expression patterns of the encoded transcript. Expression of a subset of 22q11 genes (*Dgcr8*, *Ufd1*, *Ranbp1*, Comt, *Cldn5*, *Trmt2a*, *Txnrd2*, *Cldn5, Cdc45*) is selectively enhanced in the VZ/SVZ (**inset**, top right, each panel). The color code (bottom) indicates the diverse functional classes to which each of these 22q11 genes localized to the VZ/SVZ and CNgV belong (top, after [75]; images from Genepaint; functional category key from [124]).

To assess potential influence of 22q11 gene dosage on neural progenitors, we first asked whether one, some, or all of the murine orthologues of the 32 genes in the minimal critical deleted region of human chromosome 22 whose heterozygous deletion results in the full spectrum of 22q11DS phenotypes [120] are expressed in neural precursors in the developing mouse CNS or PNS. A variety of observations using quantitative PCR, in situ hybridization, and immunolocalization indicate that a fairly substantial number of the 22q11 deleted genes (at least 22 of the full set of murine orthologues) are expressed throughout the developing nervous system, including in neural progenitors in the cerebral cortex, spinal cord, and neural crest [3, 4, 121–123]. Indeed, subsequent analyses from several laboratories [124] as well as assessment of images from Genepaint (Fig. 2), a publicly available in situ localization database [125], indicate that multiple 22q11 genes are expressed both in the ventricular and subventricular zone (VZ/SVZ) of the developing cortex as well as in cranial sensory ganglia including the trigeminal ganglion (CNgV) which is derived from both neural crest-derived and cranial placode-derived precursors [126]. Thus, multiple 22q11 genes are expressed in neural progenitors and may influence their capacity to proliferate as well as generate new neurons.

The diverse functional identities of the 22q11 genes (see Fig. 2) suggest an additional level of complexity of precursor regulation. Of the 32 minimal critical deleted region genes, plus 20 additional genes in the larger 3 MB region more commonly deleted in 22q11DS [120, 127], none are direct cell cycle regulators, although some like *Hira* [128], *Cdc45l* [129], *Ranbp1* [15], and *Trmt2a* [130] have been functionally implicated in control of progenitor proliferation. Most protein-coding 22q11 deleted genes fall into categories not specifically related to progenitor regulation: these include six mitochondrially localized genes [131], a limited number of transcription factors including *Tbx1*, a suggested 22q11DS “candidate” gene that may regulate cardiac as well as neural differentiation [132, 133], modulators of chromatin integrity and DNA repair, and several cell-cell or intracellular signaling mediators and adhesion molecules [124]. Thus, many 22q11 genes could influence progenitor progression; however, their dosage-dependent influence in the context of polygenic 22q11 deletion may be subtle, probabilistic and rely upon mechanisms that transcend uniform effects on defined progenitor types. To begin to determine whether and how genetic disruptions alter neural progenitors in the context of NDDs – one of Verne’s major goals and that of his many trainees and collaborators – we analyzed the consequences of multigenic heterozygous 22q11 gene deletion for cortical neurogenesis as well as genesis of CNgV sensory neurons (see Fig. 1).

## 22q11 Deletion Disrupts Central and Peripheral Neurogenesis

**Fig. 3.**
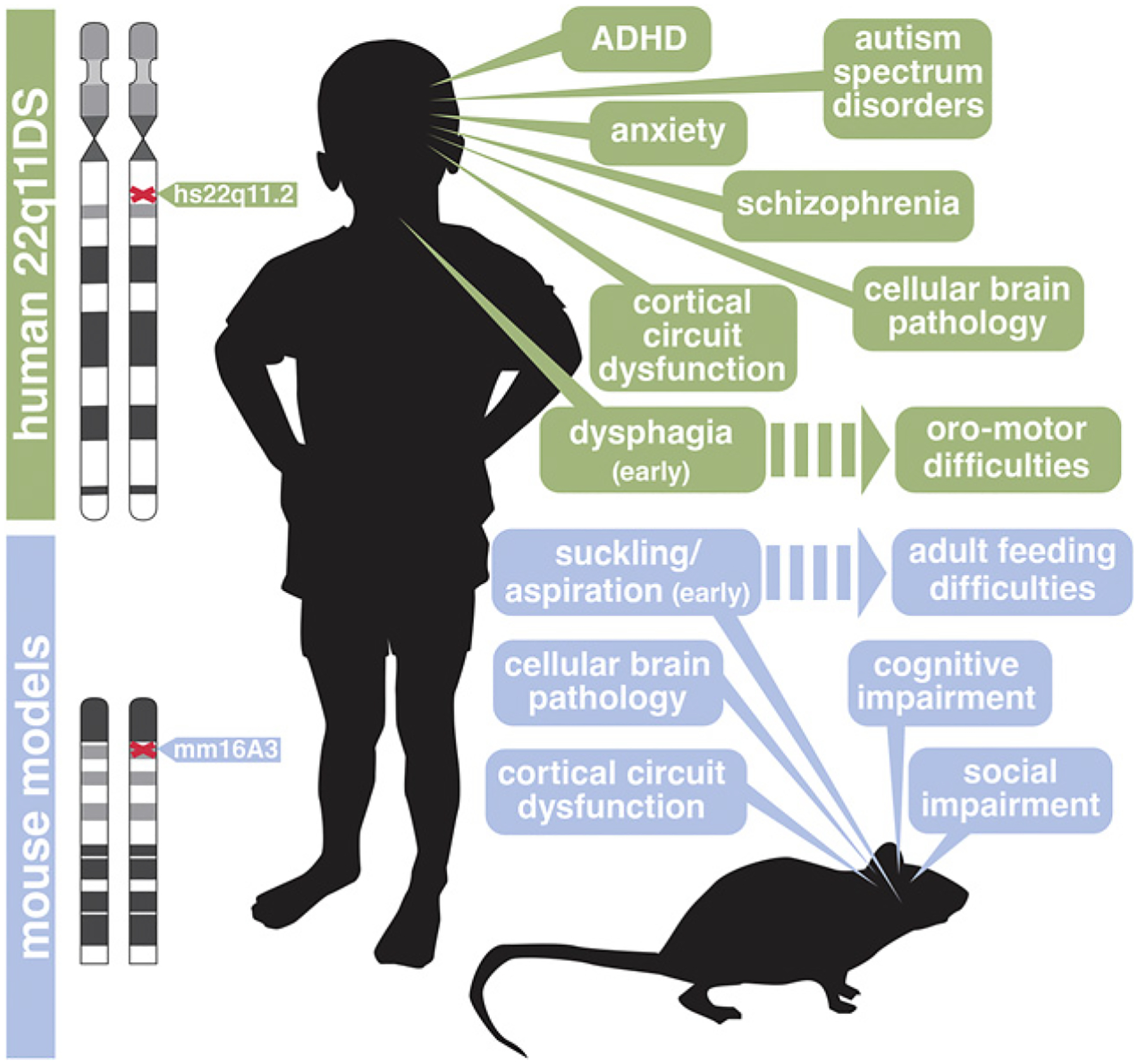
Heterozygous deletion at human chromosome 22q11 and parallel heterozygous deletion of contiguous murine orthologues on mouse chromosome 16 results in behavioral disruption and cellular pathology associated with neurodevelopmental disorders (NDDs). Top left: the location of the heterozygous deletion proximal to the centromere on the q arm of chromosome 22 that is associated with 22q11DS. Top right: the phenotypic spectrum of 22q11DS includes substantially elevated frequency of behavioral disorders that parallel clinically defined NDDs including ADHD, autism spectrum disorders (ASD), schizophrenia (Scz), and anxiety disorder. Functional imaging studies of individuals with 22q11DS indicate cortical circuit dysfunction, and structural imaging as well as a limited postmortem analyses have found likely foci of cellular brain pathology. In addition to clinically diagnosed NDDs associated with cortical circuitry and function, infants and toddlers with 22q11DS have perinatal dysphagia – difficulties with suckling, feeding, and swallowing – followed by oromotor difficulties including aberrant food ingestion, swallowing, and speech throughout the lifespan. Bottom left: location of heterozygous deletion of contiguous 22q11 orthologues that define the minimal critical deleted region associated with 22q11DS on mouse chromosome 16. Bottom right: although it is not possible to identify behavioral states in mice that fully parallel those in ADHD, ASD, Scz, or anxiety disorder, *LgDel* mice with a deletion on mmChr16 orthologous to that on hChr 22 have a spectrum of behavioral deficits and changes in cortical as well as peripheral neural circuits that can be related to aspects of human clinical disorders seen in individuals with 22q11DS throughout the lifespan (figure modified from [75]).

The first question we asked was whether heterozygous deletion of murine orthologues of human genes associated with essential diagnostic 22q11DS phenotypes (Fig. 3) (thus, the genes in the 1.5 MB minimal critical deleted region; [14]) compromises CNS or PNS neural progenitor proliferation. We also asked whether such disruptions result in altered frequency, proportions, or identities of neurons generated from these progenitor populations. Mice with heterozygous deletions genomically equivalent to those in 22q11DS have behavioral deficits (see Fig. 3) that can be related to those seen in individuals with 22q11DS as well as in clinically defined NDDs including ID, Scz, and ASD (as reviewed in [75]). Thus, using 22q11 mouse models, it may be possible to assess critically whether polygenic disruption alters CNS or PNS neural progenitor proliferation or the capacity of these precursors to generate neurons that contribute to specific circuits whose dysfunction underlies NDD pathology.

**Fig. 4.**
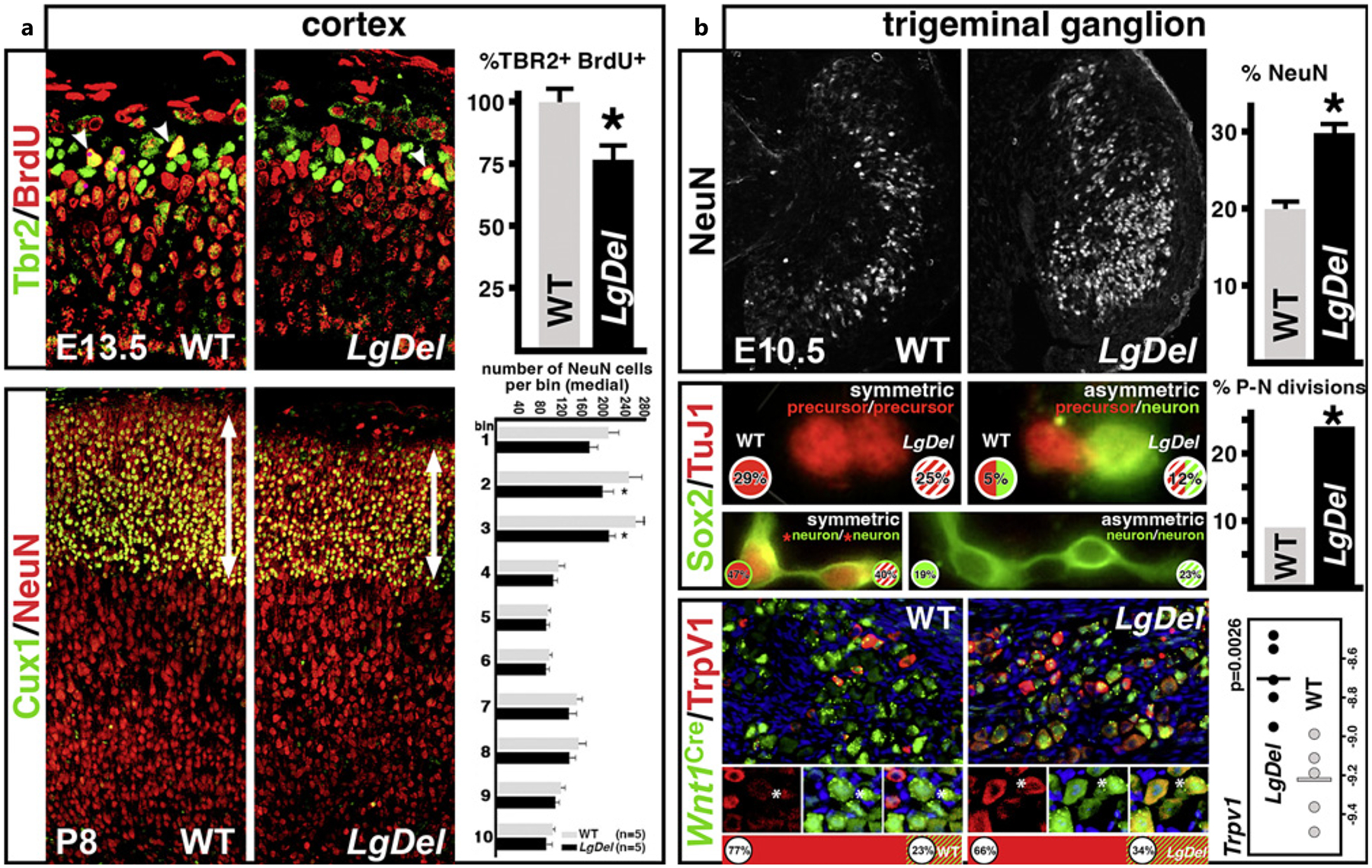
CNS and PNS neural progenitors are compromised by heterozygous deletion of murine orthologues of human 22q11 genes. **a** Selective disruption of basal progenitor proliferation during cortical histogenesis by heterozygous deletion of murine 22q11 orthologues. Top right: in E13.5 *LgDel* fetuses, as the initial population of basal progenitors is being generated, there is a diminished frequency of Tbr2+ basal progenitors that can also be labeled acutely with BrdU, indicating active proliferation. Bottom right: in neonatal *LgDel* mice (postnatal day P8), the frequency of layer 2/3 PNs, labeled selectively for Cux1 as well as the general neuronal marker NeuN (RbFox3), declines significantly; however, the frequency of NeuN+ or Ctip2+ neurons (a selective marker for layer 5/6 PNs) in layer 5/6 is not substantially changed. **b** Disrupted neurogenesis and progenitor proliferation during trigeminal ganglion (CNgV) differentiation due to heterozygous deletion of murine 22q11 orthologues. Top panels: there is an apparent increase in the frequency of newly generated neurons, labeled with NeuN, in the E10.5 *LgDel* CNgV. Middle panels: increased frequency of early generated CNgV neurons in *LgDel* apparently reflects increased symmetric neuron-neuron as well as increased asymmetric precursor-neuron divisions of neural crest-associated (Sox2+) CNgV neural progenitors. These data were generated using a pair-cell assay in which CNgV cells are dissociated, plated in microwells at very low density, and allowed to divide for 21 h. Isolated pairs of cells are presumed to derive from individual progenitors, and their identities are then probed with specific progenitor markers (in this case Sox2) as well as neuronal markers (in this case, βIII-tubulin). Bottom panels: the apparent frequency of subclasses of TrpV1+ nociceptive sensory neurons, particularly those labeled for TrpV1 and via recombination driven by Wnt1 to mark a subpopulation of neural crest-derived progenitors at earlier stages, increases in P8 *LgDel* CNgV. This increase is paralleled by increased abundance of TrpV1 mRNA in *LgDel* versus WT, measured by qPCR in samples of dissected P8 CNgV (**a** adapted from [3]; **b** from [16]).

We focused first on cortical progenitors in the VZ/SVZ of the developing cortical mantle (see Fig. 1c) in the *LgDel* mouse 22q11DS model as well as related single gene mutations. This focus seemed critical because of the robust association of 22q11DS with vulnerability for clinically defined disorders of cortical circuit development including Scz and ASD that are thought to compromise PN progeny of VZ/SVZ progenitors [134, 135]. We found that the frequency of proliferating basal progenitors in the SVZ was selectively diminished in *LgDel* fetuses at midgestation [3] (Fig. 4a, top). Additional experiments suggested that the proliferative capacity of VZ (apical) precursors was not appreciably disrupted. Our observations suggest that basal progenitors are generated by apical progenitors at similar frequencies in the *LgDel*, and the proliferative capacity of a subset is altered subsequently. The selective decrease in frequency of actively proliferating basal progenitors indicated that the frequency of their primary progeny: layer 2/3 PNs might also be selectively diminished compared to their primarily apical precursor-derived layer 5/6 PN counterparts. This was indeed the case (Fig. 4a, bottom). The overall frequency of NeuN (RbFox3) labeled neurons was diminished in layers 2/3 but not layers 4, 5, or 6. Moreover, there was a diminished frequency of presumed layer 2/3 PNs, labeled by Cux1, but not of layer 4, 5, or 6 PNs identified by Tbr1, a marker for these cells.

To better formulate hypotheses regarding the significance of selectively disrupted cortical neurogenesis in divergent neural circuit development underlying NDD pathology [32, 136], we asked whether partial depletion of layer 2/3 PNs, apparently reflecting randomly diminished proliferation of a subset of basal progenitors, might contribute to functional deficits later in life that reflect altered association cortico-cortical connections, presumably made by layer 2/3 PNs. One such deficit, the performance of a visual reversal learning task, relies critically upon association cortico-cortical connections between the medial frontal and lateral entorhinal cortex [137, 138]. In a cohort of individual *LgDel* and WT control littermate mice, we assessed the correlation between performance of this layer 2/3 PN connection-dependent task and the frequency of Layer 2/3 PNs versus that of parvalbumin-labeled interneurons (also altered in *LgDel* mice [3, 139]) in medial frontal association cortex in each individual mouse [140]. Diminished medial frontal association cortex layer 2/3 PN frequency in individual mice was highly correlated with the magnitude of reversal learning deficit [140]. In contrast, the frequency of parvalbumin-labeled interneurons does not correlate with visual reversal performance in individual mice. These results suggest that selective disruption of basal progenitor proliferation, followed by decreased frequency and perhaps divergent specification of layer 2/3 PNs, may contribute to altered association cortical circuit and related behavioral deficits due to 22q11 deletion.

Neural circuit and behavioral disruption in NDDs including 22q11DS are not confined to the cerebral cortex and its functional capacities or even the CNS [122, 141, 142]. We found that neonatal *LgDel* mice have signs of altered suckling, feeding, and swallowing (S/F/S; see Fig. 3) that parallel perinatal dysphagia in infants with 22q11DS [122, 126, 143]. Based upon the essential role of cranial sensory ganglia, particularly CNgV, in S/F/S oropharyngeal function, we asked whether neural progenitors that yield multiple classes of CNgV sensory neurons are compromised in developing *LgDel* mice. We analyzed cranial placode- and neural crest-derived neural progenitors (see Fig. 1d; [62]) at midgestation, right after CNgV has coalesced as a recognizable ganglion (see Fig. 2). There is a decline in the frequency of neural crest progenitors [16] and a proportional increase in placode-derived progenitors in the *LgDel* CNgV (Fig. 4b). This disruption is accompanied by a change in neighbor relations of placodal as well as neural crest progenitors. In the *LgDel* CNgV, placode cells are more likely to have a placode neighbor, while neural crest cells are less likely to have neural crest neighbors. These changes suggest that optimal local cell-cell signaling as well as neurogenic capacities of subsets of CNgV progenitors may be altered by heterozygous 22q11 deletion.

To explore this possibility, we asked whether the timing and frequency of neurogenesis changes in the *LgDel* CNgV. In typically developing CNgV, placodal progenitors divide more frequently early to generate mechanosensory neurons, while neural crest progenitors divide more at a slightly later time to generate nociceptive neurons [144, 145]. We found an increase in the frequency of postmitotic neurons in the *LgDel* CNgV at midgestation, accompanied by an apparent decrease in the proportion of presumed nociceptive neurons in the early postnatal ganglion (Fig. 4b, top) [16]. The early increase could reflect a greater proportion of placode-derived neurons or, counterintuitively, increased frequency of neuronal progeny of neural crest precursors generated at earlier times. This latter possibility may be the case since *LgDel* CNgV neural crest-derived progenitors yield more frequent asymmetric neurogenic divisions at the apparent expense of self-renewing progenitor divisions (Fig. 4b, middle). This change could explain several differences in the *LgDel* CNgV. First, the proportion of proliferating placode-derived progenitors would appear higher because the pool of self-renewing neural crest progenitors is diminished. Second, placode-derived progenitors would likely be more aggregated since the addition and presumably intercalation of additional neural crest progenitors is diminished. Third, altered proportions of mechanosensory versus nociceptive neurons would reflect diminished nociceptive neurogenesis at later stages of CNgV development due to a depleted progenitor pool. Thus, 22q11 deletion could alter proportions of subsets of CNgV sensory neurons by altering neighbor relationships and modes of division. These changes may reflect differences in progenitor state rather than identity: affected subsets of neural crest progenitors retain expression of established molecular markers. Due to probabilistically altered cell-cell interactions, however, their proliferative properties diverge from those of others in the same lineage.

The potential consequences of these changes among subsets of *LgDel* CNgV progenitors may include altered axon growth and guidance for the peripheral and central branches of CNgV neurons during initial ganglion differentiation [122, 146] as well as changes in proportions, sizes, and distribution of CNgV neurons with molecular characteristics of mechanoreceptive as well as nociceptive sensory neurons (Fig. 4b, bottom) [16]. These disruptions of CNgV differentiation suggest that altered interactions between subsets of placode and neural crest progenitors due to 22q11 deletion may impact oropharyngeal sensory function. We have shown that newborn *LgDel* mice have S/F/S-related difficulties [122, 147] and that feeding behaviors continue to be altered in adult *LgDel* mice [143]. We have not yet established definitive evidence of a causal connection between early CNgV neural progenitor disruption, premature neurogenesis, quantitative changes in CNgV mechanosensory and nociceptive neurons, and oropharyngeal dysfunction in S/F/S. Nevertheless, changes in nociceptive versus mechanoreceptive sensory neuron identities could disrupt peripheral sensory feedback that controls milk intake early in life and successful ingestion of solid food later in the lifespan.

## Up- and Downstream of Diminished Dosage: 22q11 Genes as Regulators of Progenitor States

The two instances of altered neural progenitor proliferation in the *LgDel* 22q11DS mouse model indicate that multigenic, dosage-dependent alterations alter neurogenic capacity of some – but not all – progenitors within molecular marker-defined subclasses. An essential question is how these disruptions result from dosage changes of 22q11 genes expressed in CNS and PNS progenitors (see Fig. 2; [4, 131]). We and others have approached this question by analyzing the consequences of loss- or gain-of-function of individual 22q11 genes as well as characterizing molecular changes that are likely downstream of diminished multigenic 22q11 gene dosage. Our results suggest that a combination of changes in mechanisms that regulate cell proliferation as well as more global changes in transcriptional regulation modify neural progenitors in the context of heterozygous 22q11 gene deletion.

CNV disorders are often analyzed as “contiguous gene syndromes” in which individual genes within a larger deleted or duplicated chromosomal region are associated with distinct phenotypes. Our functional genomic, informatic, and literature-based assessments of genes within the 1.5 MB 22q11DS minimal critical deleted as well as the more common 3.0 MB typically deleted region suggest that none of the 22q11 genes regulate primarily cell cycle or cytokinesis [3, 4, 124]. Nevertheless, several of the 22q11 deleted genes may influence neural progenitor progression, based upon expression localization (see Fig. 2) and function in pathways that can modulate cell proliferation and division in addition to other signal transduction cascades. These include the chromatin elongation/duplication regulator CDC45 and the nuclear/cytoplasmic transport regulator RANBP1. Based upon its function in chromosomal duplication during cell division as well as localization in subsets of dividing cells, CDC45 is a potential candidate for disrupted neural progenitor disruption due to 22q11 deletion. Nevertheless, potential functions of CDC45 in neural progenitors have not been explored in animal models due to early postimplantation lethality of homozygous *Cdc45* null mutations, as well as a lack of apparent phenotypes in *Cdc45* heterozygous mutant mice [148].

**Fig. 5.**
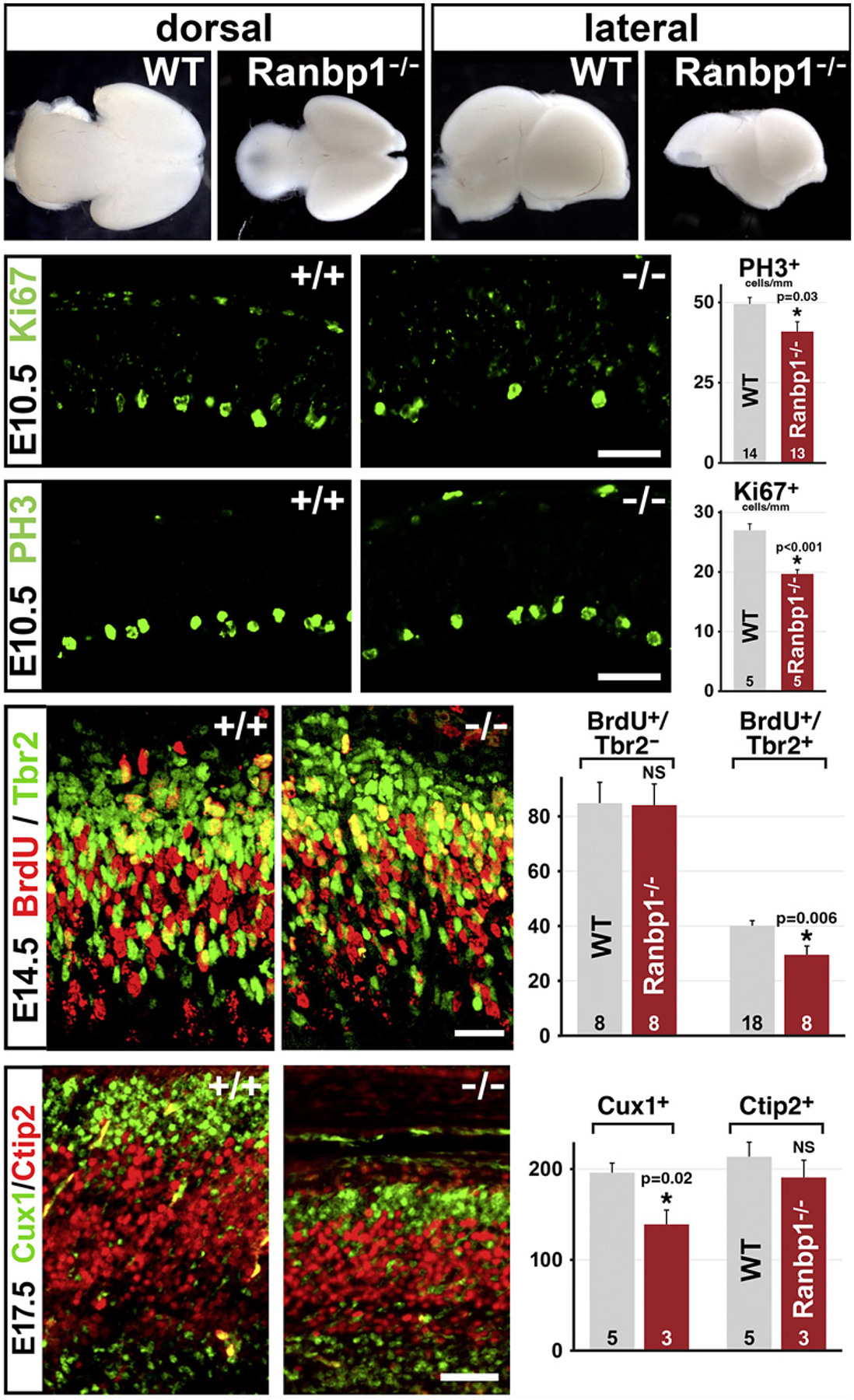
Homozygous null mutation of the 22q11 deleted gene *Ranbp1* results in microcephaly due to diminished apical and basal progenitor proliferation. Top panels: at E18.5, *Ranbp1*^−/−^ mice, who die at birth, are visibly microcephalic, based upon head size (not shown) and brain size, especially that of the cerebral hemispheres. These changes in brain size are visible both from a dorsal (left) and latral (right) view. Middle panels: at E10.5, when the cortical rudiment is still a proliferative neurepithelium with a small number of postmitotic neurons, active proliferation of neuroepithelial/apical progenitors, labeled with Ki67, is diminished in *Ranbp1*^−/−^ fetuses, as is the frequency of PH3+ presumed mitotic progenitors. Lower panels: by E14, when basal progenitor genesis from apical progenitors has begun, there are fewer Tbr2+ basal progenitors acutely labeled by BrdU in *Ranbp1*^−*/*−^ fetuses. By E17.5, when an appreciable number of layer 2/3 PNs, labeled here by Cux1, have accumulated in the WT cortex, there are significantly fewer Cux1+ layer 2/3 PNs in the *Ranbp1*^−/−^ cortex; however, the frequency of Ctip2+ layer 5/6 PNs is not significantly reduced (all panels adapted from [15]).

Among additional candidate genes, we focused upon *Ranbp1* as a potential regulator of basal progenitor function based upon its high level of expression in the SVZ and its modulation of signaling that can influence cell cycle progression and cell division [149]. We developed a mouse model with a null mutation of the *Ranbp1* gene [15]. Full *Ranbp1* loss-of-function is lethal around birth and has a variety of developmental consequences including acting as an apparent microcephaly gene [146]. Gross microcephaly is seen in late gestation *Ranbp1*^−*/*−^ fetuses. The reduced size of the cerebral cortex reflects disruptions of both early apical (Fig. 5, middle) and later basal progenitor populations (Fig. 5, bottom). The consequences for the basal progenitor population appear most substantial: layer 2/3 PN numbers are dramatically depleted (Fig. 5, bottom) so that cortical thickness as well as cortical size is diminished. The *Ranbp1*^−/−^ cortical phenotype suggests that heterozygous deletion of *Ranpb1*, acting as a contiguous gene, may account for cortical progenitor phenotypes seen with broader heterozygous 22q11 deletion. Basal progenitor proliferation, however, does not change significantly in heterozygous *Ranbp1+/*− mutants [146]. Thus, diminished basal progenitor proliferation in *LgDel* likely reflects dosage-dependent interactions between *Ranbp1* and additional 22q11 genes with diverse expression and functions in cortical progenitors (see Fig. 2). Disruption of these interactions by 22q11 deletion is likely to contribute to aberrant differentiation of layer 2/3 PNs as well as related behavioral deficits [75, 150].

**Fig. 6.**
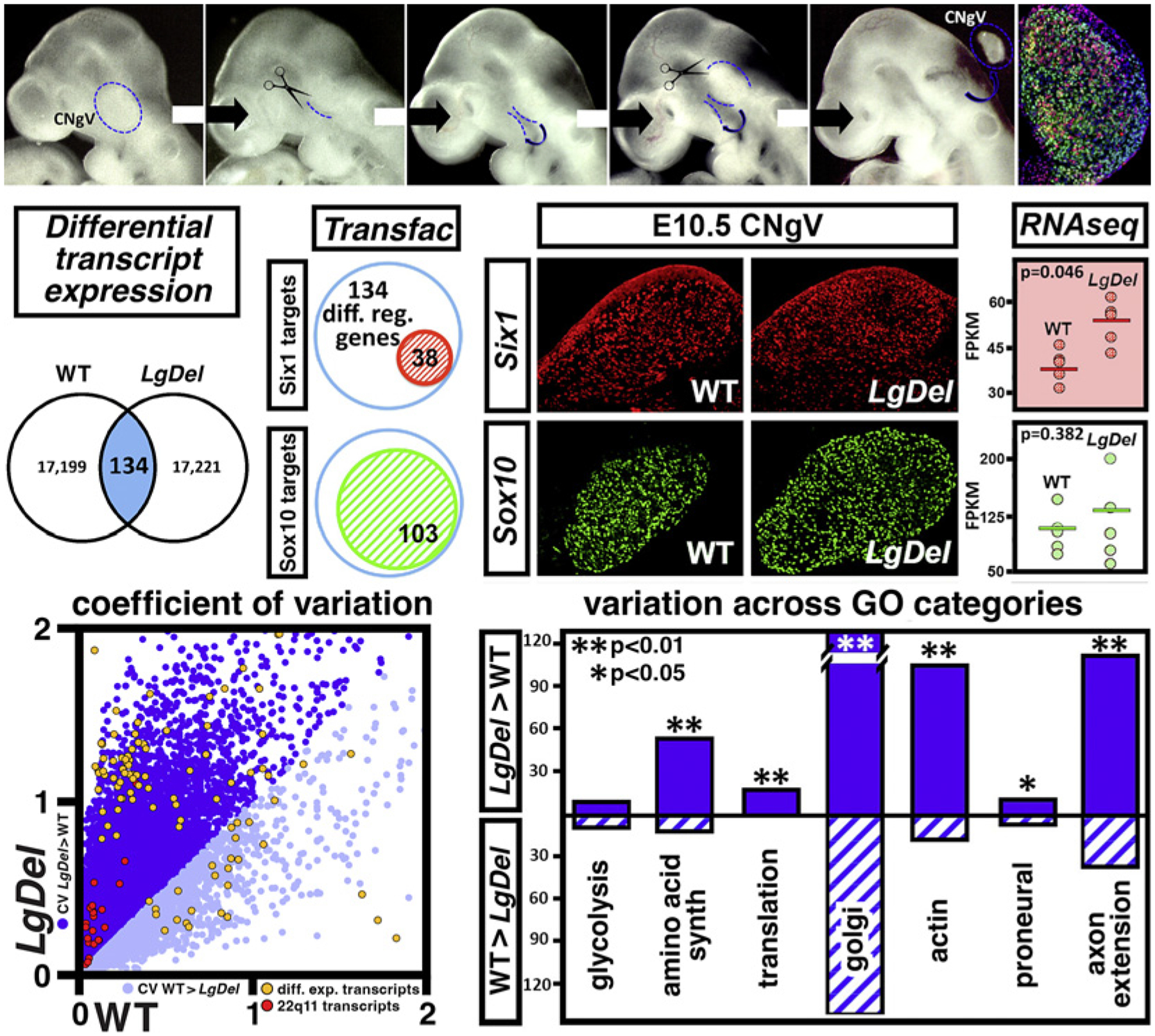
Transcriptional divergence of E10.5 *LgDel* CNgV neural progenitors and newly generated neurons. Top panels: we dissected CNgV from WT and *LgDel* E10.5 fetuses, an age when substantial numbers of placodal (Six1+, red) and neural crest (*Wnt1*^Cre-^recombined + or −, green and blue, respectively) can be seen in the ganglion (far right). These dissected CNgV from multiple embryos and multiple litters were pooled to collect 5 biological replicates for RNAseq transcriptome analysis. Middle panels: comparison of the 5 biological replicate CNgV transcriptomes identified 134 genes that are differentially expressed in *LgDel* versus WT, based upon a significance threshold of FDR q < 0.1 (left). Of these 134 genes, 38 can be informatically identified as potential targets for transcriptional regulation by Six1, a diagnostic transcription factor expressed in placode-derived CNgV progenitors, and 130 can be identified as potential targets for transcriptional regulation by Sox10, a diagnostic transcription factor marker for a subpopulation of neural crest-derived CNgV progenitors (right). Lower panels: there is a significant transcriptome-wide increase in the coefficient of variation for genes expressed in the E10.5 *LgDel* versus WT CNgV. Increased variation is seen for 22q11 genes whose expression is detected in the E10.5 CNgV (red dots) as well as the 134 genes differentially expressed between *LgDel* and WT (orange dots). Increased variation can be seen across functional categories (right) that include fundamental cellular metabolic and protein/membrane trafficking processes (amino acid synthesis and Golgi) as well as those specific for neural progenitors and differentiating neurons (proneural genes, axon extension genes). Asterisks indicate significance levels as shown in the top left corner (all panels adapted from [123]).

The impact of diminished dosage of multiple 22q11 genes on transcriptional regulation that ultimately defines neural progenitor proliferative and neurogenic capacities is not yet clear. To begin to address this question, we analyzed the transcriptomes of *LgDel* and WT CNgV at E10.5 (Fig. 6, top), when there is peak proliferation of both placode- and neural crest-derived progenitors in the nascent ganglia (see Fig. 5 and [62]). We assessed differential gene expression in WT versus *LgDel* CNgV (Fig. 6, middle right). We identified differentially expressed genes among the approximately 17,200 significantly expressed transcripts shared by WT and *LgDel* CNgV by comparing mean expression levels of each across 5 WT and *LgDel* replicate transcriptome samples [123]. There is a relatively modest number – 134 (0.7% of total shared transcripts) – of significantly differentially expressed genes, including a subset of 22q11 genes whose expression diminishes by the expected 50%. The range of fold changes – increase or decrease – is substantial: from nearly 200 to less than 2-fold. The absolute expression levels of most differentially expressed transcripts, however, are modest. Moreover, there is no single functional signature among the group. We further assessed the association of the differentially expressed genes with progenitor classes whose identity is defined by distinct transcription factors (Fig. 6, middle right). Based upon informatic genomic sequence analysis, most differentially expressed loci have binding sites for Six1 (placode), as well as Sox10 and FoxD3 (neural crest subpopulations) [62, 151, 152]. In contrast, no differentially expressed genes were informatically associated with Brn3a, a transcription factor seen in differentiating CNgV neurons. It is possible that levels of differential CNgV gene expression reflect distinct transcriptional regulation of the same genes in placodal versus neural crest progenitors.

In addition to diversity of transcriptional regulation of common targets, expression levels in the *LgDel* versus WT CNgV appear to vary more across the entire transcriptome, despite presumed reduction of variability realized by pooling samples across embryos and litters [123]. To quantify this impression, we determined the coefficient of variation for each of the 17,000+ shared transcripts and found that approximately 15,000 (88%: including several 22q11 and other differentially expressed genes) had a greater coefficient of variation in *LgDel* than WT (Fig. 6, bottom left). Moreover, expression levels of transcripts from gene ontology categories to which the differentially expressed genes belong are significantly more variable in *LgDel* CNgV (Fig. 6). These data suggest that progenitor identities reflect transcript levels, rather than singular expression or repression, of a broad spectrum of genes. When diminished dosage of 22q11 genes disrupts these quantitatively defined transcriptional states, the proliferative characteristics and neurogenic capacity of individual progenitors within a quantitatively diverse population may be destabilized. These potentially stochastic changes in variable expression independent of progenitor types identified by unique markers could result in changes in number of neurons generated as well as the capacity of postmitotic progeny to differentiate optimally.

## Lining Up for Altered States: Balancing Progenitor Identities with Neurogenic Flexibility

Among the complexities of cortical neurogenesis that Verne recognized in the 1970s and explored in depth over the subsequent four decades, he identified two fundamental characteristics of neural progenitors that remain central for understanding brain development and its disruption by NDDs. First, broad molecular identities of neural progenitors (i.e., those resolved by cell class-specific markers or distinct cell cycle times) vary, indicating lineage constraints. Second, additional variables must contribute to the diversity of neuron types these progenitors generate. The vulnerability of cortical neurogenesis, and indeed neurogenesis more broadly, to genetic mutation and environmental disruptions that underlie NDDs may reflect, at least in part, the second class of variables, i.e., those that establish progenitor states rather than progenitor classes. These mechanisms may regulate the amount of variability among lineally related progenitors belonging to apparently singular classes, leading to as yet unrecognized neurogenic diversity or flexibility. NDD-associated mutations may modify or destabilize signaling, cell cycle phases, or modes of division for subsets of otherwise similar progenitors and thus alter numbers of neuronal progeny or their capacity for optimal differentiation.

**Fig. 7.**
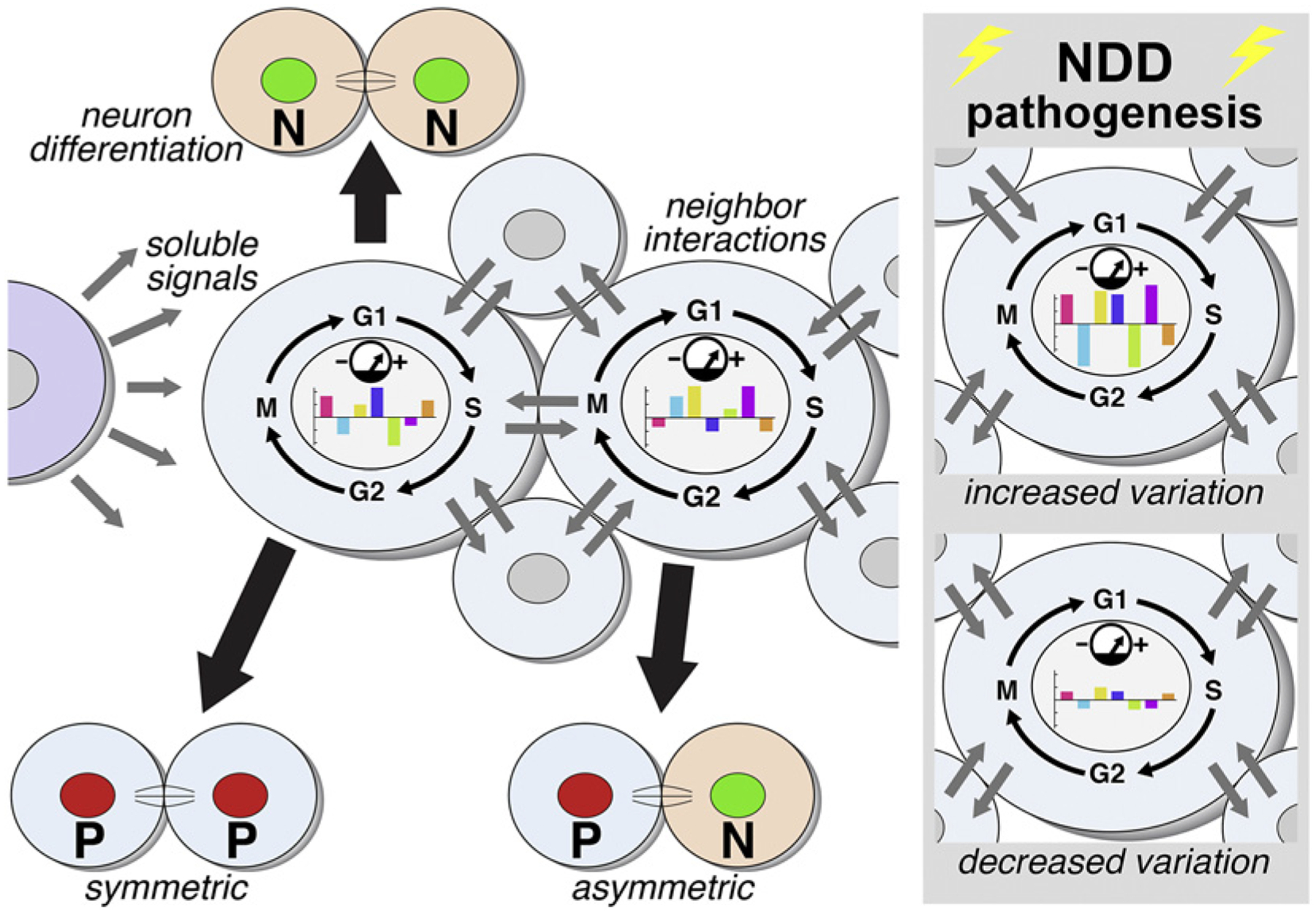
State-dependent regulation and diversification of neural progenitor classes whose broad identities are defined by molecular markers. These progenitors, of which two examples are shown here in detail, retain the unique expression of their diagnostic molecular marker, often reflecting derivation from another broad class of progenitors. Their transcriptional identities (indicated here by the colored bars on the histograms within the nuclei of the two cells) are at one level of analysis – absolute presence of multiple transcripts – equivalent. Their states, however, are divergent based upon variation of levels of expression of the set of transcripts shared by the two otherwise indistinguishable progenitors. These states, indicated here based upon levels of transcription of identified genes, are likely established by diffusible extrinsic signals as well as signaling that relies upon cell-cell contact. The targets of these signals for modifying progenitor state likely include activation or repression of signaling pathways that influence transcription as well as chromatin regulation via direct DNA methylation or changes in histone modifications. NDD pathogenic processes may target these state-modulation mechanisms without changing broad identities of molecular marker-defined progenitor classes. The consequences of these “altered states” would be recognized as increases or decreases in the transcription variability due to quantitative alteration of signaling pathways and their downstream targets including posttranslational modification of transcriptional regulators as well as chromatin-modifying enzymes.

In the decades following the 1978 landmark review by Caviness and Rakic, great insight into the first issue of broad progenitor classes, their proliferative characteristics, and lineage constraints has been generated by identifying a number of selective markers for neural progenitors in distinct brain regions [95, 153–156], analyzing flexibility as well as limitations of precursor capacities and progeny fates using real-time imaging in vitro [72, 157, 158] and by studying single gene mutations that compromise CNS or PNS progenitors [2, 159–164]. Nevertheless, it remains difficult to map this still fairly limited number of molecularly distinct neural progenitor types onto the diversity of postmitotic neuronal progeny in any CNS or PNS region. The extensive literature on Notch-related signaling [55] as well as signaling via Wnt-β catenin [165] and other pathways [64, 65, 166] in neural progenitors suggests that these signals contribute to variation that modifies the states of neural progenitors without changing their membership in broadly defined progenitor classes. Small variations in local soluble or secreted signals may also add to this state-dependent variability (Fig. 7). For example, the cilium of one cortical precursor might access sufficient concentration of Sonic hedgehog [65, 166] to elicit signal transduction and a subsequent transcriptional response, while that of its near neighbors may not. Such local divergence could account for significant additional variation among otherwise molecularly similar progenitors and their progeny; its disruption could have substantial consequences for neurogenesis and neural circuit differentiation.

Recent analysis of progenitor subpopulations based upon single-cell transcriptomes [167–171] from the developing cortex suggests substantial quantitative heterogeneity [168, 169, 172–174]. In comparisons of sorted cortical progenitor types, single molecular marker-defined populations have dynamic, quantitatively diverse transcriptional signatures [170]. Regulation via miRNAs [175, 176] and other RNA-mediated processes [177] may contribute to progenitor diversity. Dynamic changes in chromatin conformation could further diversify neural progenitor states [178–181] and thus increase flexibility of neuronal differentiation. Some chromatin modifications may be transient, depending upon local interactions, cell cycle phases, and the abundance and activity in individual progenitors of proteins that regulate DNA methylation [182, 183] or histone modifications [171, 180]. Indeed, variation in the sequence or quantity of chromatin modifications among different progenitors with the same broad molecular identity may provide flexibility for increasing the possible range of progenitor states that bias postmitotic neuroblasts toward a broader spectrum of differentiated neuron types. The durability of DNA methylation or histone modification and how these chromatin marks are transferred to the progeny of individual progenitors is likely key for relating the proliferative history to the differentiation capacity of any neuron. The sequential accumulation or removal of direct DNA methylation or specific histone modifications may “keep time” for individual neural progenitors and provide a history of signaling, division, and transcriptional states as a foundation for a distinct program of differentiation for postmitotic progeny. For example, divergent DNA methylation in a subset of apical or basal cortical progenitors could bias differentiation of apparently similar progeny to PN subclasses that share a general laminar destination and similar time of final division but have distinct axonal targets, synaptic inputs, and functional signatures. This epigenetic adaptability, however, may come with a price: dynamic and flexible epigenetic progenitor proliferation could easily be disrupted by multiple genetic or environmental insults associated with NDD pathology. Increased or decreased transcriptional variability among neural progenitors due to NDD-related pathogenic mechanisms that target chromatin modification could substantially alter neurogenesis and subsequent neuronal differentiation (Fig. 7, right).

## Conclusion

The two examples of 22q11 deletion-mediated disruption of neural progenitor proliferation and genesis of related neuron classes in 22q11DS mouse models provide a starting point to consider how molecularly identified precursor types that get “in line” can be further diversified to yield highly flexible progenitors, each in a distinct “state” based upon quantitative differences in signaling and epigenetic modifications (Fig. 7). During cortical neurogenesis, 22q11DS compromises basal progenitors as a broad class; however, it is not clear whether changes are targeted to a molecularly distinct basal progenitor subset. These changes may be random, due to 22q11 gene dosage-dependent modulation of signaling, modes of division, epigenetic modification, and transcriptional signature of individual progenitors. The robust, apparently mosaic, expression of multiple 22q11 genes in cortical and CNgV progenitors may further vary due to stochastic allelic regulation [184]. Thus, even in neighboring cells, expression levels of heterozygously deleted 22q11 genes may rise below or above the presumed 50%, creating a matrix of progenitors with effectively null to wild-type levels or beyond. These variable 22q11 gene dosages among single cells may initiate additional pathogenesis among otherwise apparently equivalent progenitors. Such 22q11 gene-dependent “altered states” may diminish proliferation for some progenitors, alter modes of division for others, and modify the differentiation capacity of subsets of layer 2/3 PNs or CNgV sensory neurons. Downstream of 22q11 gene dosage, local signaling including that leading to epigenetic modifications may vary beyond adaptive limits, changing a spectrum of optimal progenitor states to a mosaic of suboptimal states that compromise circuit development, organization, and behavior in 22q11DS [75, 150, 185–187].

## References

[1] Rakic P, Caviness VS Jr. Cortical development: view from neurological mutants two decades later. Neuron. 1995 Jun;14(6):1101–4. 7605626 10.1016/0896-6273(95)90258-9

[2] Caviness VS Jr, Rakic P. Mechanisms of cortical development: a view from mutations in mice. Annu Rev Neurosci. 1978;1:297–326. 386903 10.1146/annurev.ne.01.030178.001501

[3] Meechan DW, Tucker ES, Maynard TM, LaMantia AS. Diminished dosage of 22q11 genes disrupts neurogenesis and cortical development in a mouse model of 22q11 deletion/DiGeorge syndrome. Proc Natl Acad Sci U S A. 2009 Sep 22;106(38):16434–45. 19805316 10.1073/pnas.0905696106 PMC2752572

[4] Maynard TM, Haskell GT, Peters AZ, Sikich L, Lieberman JA, LaMantia AS. A comprehensive analysis of 22q11 gene expression in the developing and adult brain. Proc Natl Acad Sci U S A. 2003 Nov 25;100(24):14433–8. 14614146 10.1073/pnas.2235651100 PMC283609

[5] Herrup K. Role of staggerer gene in determining cell number in cerebellar cortex. I. Granule cell death is an indirect consequence of staggerer gene action. Brain Res. 1983 Dec;313(2):267–74. 6667376 10.1016/0165-3806(83)90225-0

[6] Lee EY, Chang CY, Hu N, Wang YC, Lai CC, Herrup K, Mice deficient for Rb are nonviable and show defects in neurogenesis and haematopoiesis. Nature. 1992 Sep 24;359(6393):288–94. 1406932 10.1038/359288a0

[7] Wetts R, Herrup K. Interaction of granule, Purkinje and inferior olivary neurons in lurcher chimeric mice. II. Granule cell death. Brain Res. 1982 Nov 4;250(2):358–62. 7171994 10.1016/0006-8993(82)90431-0

[8] Gandal MJ, Haney JR, Wamsley B, Yap CX, Parhami S, Emani PS, Broad transcriptomic dysregulation occurs across the cerebral cortex in ASD. Nature. 2022 Nov;611(7936):532–9. 36323788 10.1038/s41586-022-05377-7 PMC9668748

[9] Hettwer MD, Lariviere S, Park BY, van den Heuvel OA, Schmaal L, Andreassen OA, Coordinated cortical thickness alterations across six neurodevelopmental and psychiatric disorders. Nat Commun. 2022 Nov 11;13(1):6851. 36369423 10.1038/s41467-022-34367-6 PMC9652311

[10] Shenton ME, Dickey CC, Frumin M, McCarley RW. A review of MRI findings in schizophrenia. Schizophr Res. 2001 Apr 15;49(1–2):1–52. 10.1016/s0920-9964(01)00163-3 PMC2812015 11343862

[11] Chakrabarti L, Galdzicki Z, Haydar TF. Defects in embryonic neurogenesis and initial synapse formation in the forebrain of the Ts65Dn mouse model of Down syndrome. J Neurosci. 2007 Oct 24;27(43):11483–95. 17959791 10.1523/JNEUROSCI.3406-07.2007 PMC6673208

[12] Ferland RJ, Batiz LF, Neal J, Lian G, Bun-dock E, Lu J, Disruption of neural progenitors along the ventricular and subventricular zones in periventricular heterotopia. Hum Mol Genet. 2009 Feb 1;18(3):497–516. 18996916 10.1093/hmg/ddn377 PMC2722192

[13] Yabut O, Domogauer J, D’Arcangelo G. Dyrk1A overexpression inhibits proliferation and induces premature neuronal differentiation of neural progenitor cells. J Neurosci. 2010 Mar 17;30(11):4004–14. 20237271 10.1523/JNEUROSCI.4711-09.2010 PMC3842457

[14] Merscher S, Funke B, Epstein JA, Heyer J, Puech A, Lu MM, TBX1 is responsible for cardiovascular defects in velo-cardio-facial/DiGeorge syndrome. Cell. 2001 Feb 23;104(4):619–29. 11239417 10.1016/s0092-8674(01)00247-1

[15] Paronett EM, Meechan DW, Karpinski BA, LaMantia AS, Maynard TM. Ranbp1, deleted in DiGeorge/22q11.2 deletion syndrome, is a microcephaly gene that selectively disrupts layer 2/3 cortical projection neuron generation. Cereb Cortex. 2015 Oct;25(10):3977–93. 25452572 10.1093/cercor/bhu285 PMC4585528

[16] Karpinski BA, Maynard TM, Bryan CA, Yitsege G, Horvath A, Lee NH, Selective disruption of trigeminal sensory neurogenesis and differentiation in a mouse model of 22q11.2 deletion syndrome. Dis Model Mech. 2022 Feb 1;15(2):15. 10.1242/dmm.047357 PMC8126478 33722956

[17] Hu WF, Chahrour MH, Walsh CA. The diverse genetic landscape of neurodevelopmental disorders. Annu Rev Genomics Hum Genet. 2014;15:195–213. 25184530 10.1146/annurev-genom-090413-025600 PMC10591257

[18] Finer NN, Robertson CM, Richards RT, Pinnell LE, Peters KL. Hypoxic-ischemic encephalopathy in term neonates: perinatal factors and outcome. J Pediatr. 1981 Jan;98(1):112–7. 7452386 10.1016/s0022-3476(81)80555-0

[19] Vannucci SJ, Back SA. The vannucci model of hypoxic-ischemic injury in the neonatal rodent: 40 years later. Dev Neurosci. 2022;44(4–5):186–93. 35263745 10.1159/000523990

[20] van Rooij D, Anagnostou E, Arango C, Auzias G, Behrmann M, Busatto GF, Cortical and subcortical brain morphometry differences between patients with autism spectrum disorder and healthy individuals across the lifespan: results from the ENIGMA ASD working group. Am J Psychiatry. 2018 Apr 1;175(4):359–69. 29145754 10.1176/appi.ajp.2017.17010100 PMC6546164

[21] Minshew NJ, Williams DL. The new neuro-biology of autism: cortex, connectivity, and neuronal organization. Arch Neurol. 2007 Jul;64(7):945–50. 17620483 10.1001/archneur.64.7.945 PMC2597785

[22] Batiuk MY, Tyler T, Dragicevic K, Mei S, Rydbirk R, Petukhov V, Upper cortical layer-driven network impairment in schizophrenia. Sci Adv. 2022 Oct 14;8(41):eabn8367. 36223459 10.1126/sciadv.abn8367 PMC9555788

[23] van Erp TGM, Walton E, Hibar DP, Schmaal L, Jiang W, Glahn DC, Cortical brain abnormalities in 4474 individuals with schizophrenia and 5098 control subjects via the enhancing neuro imaging genetics through meta analysis (ENIGMA) consortium. Biol Psychiatry. 2018 Nov 1;84(9):644–54. 29960671 10.1016/j.biopsych.2018.04.023 PMC6177304

[24] Selemon LD, Zecevic N. Schizophrenia: a tale of two critical periods for prefrontal cortical development. Transl Psychiatry. 2015 Aug 18;5(8):e623. 26285133 10.1038/tp.2015.115 PMC4564568

[25] Stoner R, Chow ML, Boyle MP, Sunkin SM, Mouton PR, Roy S, Patches of disorganization in the neocortex of children with autism. N Engl J Med. 2014 Mar 27;370(13):1209–19. 24670167 10.1056/NEJMoa1307491 PMC4499461

[26] Wegiel J, Kuchna I, Nowicki K, Imaki H, Wegiel J, Marchi E, The neuropathology of autism: defects of neurogenesis and neuronal migration, and dysplastic changes. Acta Neuropathol. 2010 Jun;119(6):755–70. 20198484 10.1007/s00401-010-0655-4 PMC2869041

[27] Kaushik G, Zarbalis KS. Prenatal neurogenesis in autism spectrum disorders. Front Chem. 2016;4:12. 27014681 10.3389/fchem.2016.00012 PMC4791366

[28] Willsey HR, Willsey AJ, Wang B, State MW. Genomics, convergent neuroscience and progress in understanding autism spectrum disorder. Nat Rev Neurosci. 2022 Jun;23(6):323–41. 35440779 10.1038/s41583-022-00576-7 PMC10693992

[29] Willsey HR, Exner CRT, Xu Y, Everitt A, Sun N, Wang B, Parallel in vivo analysis of large-effect autism genes implicates cortical neurogenesis and estrogen in risk and resilience. Neuron. 2021 Mar 3;109(8):1409–804 e8. 33887193 10.1016/j.neuron.2021.03.030 PMC8145999

[30] Rasanen N, Tiihonen J, Koskuvi M, Lehtonen Š, Koistinaho J. The iPSC perspective on schizophrenia. Trends Neurosci. 2022 Jan;45(1):8–26. 34876311 10.1016/j.tins.2021.11.002

[31] Lewis DA. Cortical circuit dysfunction and cognitive deficits in schizophrenia: implications for preemptive interventions. Eur J Neurosci. 2012 Jun;35(12):1871–8. 22708598 10.1111/j.1460-9568.2012.08156.x PMC3383640

[32] Geschwind DH, Levitt P. Autism spectrum disorders: developmental disconnection syndromes. Curr Opin Neurobiol. 2007 Feb;17(1):103–11. 17275283 10.1016/j.conb.2007.01.009

[33] Rakic P. Synaptic specificity in the cerebellar cortex: study of anomalous circuits induced by single gene mutations in mice. Cold Spring Harb Symp Quant Biol. 1976;40:333–46. 7381 10.1101/sqb.1976.040.01.033

[34] Caviness VS Jr. Patterns of cell and fiber distribution in the neocortex of the reeler mutant mouse. J Comp Neurol. 1976 Dec 15;170(4):435–47. 1002868 10.1002/cne.901700404

[35] Stanfield BB, Caviness VS Jr, Cowan WM. The organization of certain afferents to the hippocampus and dentate gyrus in normal and reeler mice. J Comp Neurol. 1979 Jun 1;185(3):461–83. 438367 10.1002/cne.901850304

[36] Mochida GH, Walsh CA. Genetic basis of developmental malformations of the cerebral cortex. Arch Neurol. 2004 May;61(5):637–40. 15148137 10.1001/archneur.61.5.637

[37] Zaqout S, Kaindl AM. Autosomal recessive primary microcephaly: not just a small brain. Front Cell Dev Biol. 2021;9:784700. 35111754 10.3389/fcell.2021.784700 PMC8802810

[38] Jayaraman D, Bae BI, Walsh CA. The genetics of primary microcephaly. Annu Rev Genomics Hum Genet. 2018 Aug 31;19(1):177–200. 29799801 10.1146/annurev-genom-083117-021441

[39] Manzini MC, Walsh CA. What disorders of cortical development tell us about the cortex: one plus one does not always make two. Curr Opin Genet Dev. 2011 Jun;21(3):333–9. 21288712 10.1016/j.gde.2011.01.006 PMC3139684

[40] Gleeson JG, Walsh CA. Neuronal migration disorders: from genetic diseases to developmental mechanisms. Trends Neurosci. 2000 Aug;23(8):352–9. 10906798 10.1016/s0166-2236(00)01607-6

[41] Phan TP, Holland AJ. Time is of the essence: the molecular mechanisms of primary microcephaly. Genes Dev. 2021 Dec 1;35(23–24):1551–78. 34862179 10.1101/gad.348866.121 PMC8653793

[42] Hong SE, Shugart YY, Huang DT, Shahwan SA, Grant PE, Hourihane JO, Autosomal recessive lissencephaly with cerebellar hypoplasia is associated with human RELN mutations. Nat Genet. 2000 Sep;26(1):93–6. 10973257 10.1038/79246

[43] Lui JH, Hansen DV, Kriegstein AR. Development and evolution of the human neocortex. Cell. 2011 Jul 8;146(1):18–36. 21729779 10.1016/j.cell.2011.06.030 PMC3610574

[44] Gleeson JG, Luo RF, Grant PE, Guerrini R, Huttenlocher PR, Berg MJ, Genetic and neuroradiological heterogeneity of double cortex syndrome. Ann Neurol. 2000 Feb;47(2):265–9. 10665503

[45] Pilz DT, Matsumoto N, Minnerath S, Mills P, Gleeson JG, Allen KM, LIS1 and XLIS (DCX) mutations cause most classical lissencephaly, but different patterns of malformation. Hum Mol Genet. 1998 Dec;7(13):2029–37. 9817918 10.1093/hmg/7.13.2029

[46] D’Arcangelo G, Miao GG, Chen SC, Soares HD, Morgan JI, Curran T. A protein related to extracellular matrix proteins deleted in the mouse mutant reeler. Nature. 1995 Apr 20;374(6524):719–23. 7715726 10.1038/374719a0

[47] Schmid RS, Jo R, Shelton S, Kreidberg JA, Anton ES. Reelin, integrin and DAB1 interactions during embryonic cerebral cortical development. Cereb Cortex. 2005 Oct;15(10):1632–6. 15703255 10.1093/cercor/bhi041

[48] Dulabon L, Olson EC, Taglienti MG, Eisenhuth S, McGrath B, Walsh CA, Reelin binds alpha3beta1 integrin and inhibits neuronal migration. Neuron. 2000 Jul;27(1):33–44. 10939329 10.1016/s0896-6273(00)00007-6

[49] Anton ES, Kreidberg JA, Rakic P. Distinct functions of alpha3 and alpha(v) integrin receptors in neuronal migration and laminar organization of the cerebral cortex. Neuron. 1999 Feb;22(2):277–89. 10069334 10.1016/s0896-6273(00)81089-2

[50] Caviness VS Jr, Nowakowski RS, Bhide PG. Neocortical neurogenesis: morphogenetic gradients and beyond. Trends Neurosci. 2009 Aug;32(8):443–50. 19635637 10.1016/j.tins.2009.05.003 PMC2725216

[51] Caviness VS, Bhide PG, Nowakowski RS. Histogenetic processes leading to the laminated neocortex: migration is only a part of the story. Dev Neurosci. 2008;30(1–3):82–95. 18075257 10.1159/000109854 PMC2712731

[52] Tarui T, Takahashi T, Nowakowski RS, Hayes NL, Bhide PG, Caviness VS. Overexpression of p27 Kip 1, probability of cell cycle exit, and laminar destination of neocortical neurons. Cereb Cortex. 2005 Sep;15(9):1343–55. 15647527 10.1093/cercor/bhi017

[53] Caviness VS Jr, Goto T, Tarui T, Takahashi T, Bhide PG, Nowakowski RS. Cell output, cell cycle duration and neuronal specification: a model of integrated mechanisms of the neocortical proliferative process. Cereb Cortex. 2003 Jun;13(6):592–8. 12764033 10.1093/cercor/13.6.592

[54] Wakamatsu Y, Maynard TM, Weston JA. Fate determination of neural crest cells by NOTCH-mediated lateral inhibition and asymmetrical cell division during gangliogenesis. Development. 2000 Jul;127(13):2811–21. 10851127 10.1242/dev.127.13.2811

[55] Chenn A, McConnell SK. Cleavage orientation and the asymmetric inheritance of Notch1 immunoreactivity in mammalian neurogenesis. Cell. 1995 Aug 25;82(4):631–41. 7664342 10.1016/0092-8674(95)90035-7

[56] Trapnell C. Defining cell types and states with single-cell genomics. Genome Res. 2015 Oct;25(10):1491–8. 26430159 10.1101/gr.190595.115 PMC4579334

[57] Llorca A, Ciceri G, Beattie R, Wong FK, Diana G, Serafeimidou-Pouliou E, A stochastic framework of neurogenesis underlies the assembly of neocortical cytoarchitecture. Elife. 2019 Nov 18;8:e51381. 31736464 10.7554/eLife.51381 PMC6968929

[58] Klingler E, Jabaudon D. Do progenitors play dice? Elife. 2020 Jan 17;9:e54042. 31951199 10.7554/eLife.54042 PMC6968926

[59] Knowles R, Dehorter N, Ellender T. From progenitors to progeny: shaping striatal circuit development and function. J Neurosci. 2021 Nov 17;41(46):9483–502. 34789560 10.1523/JNEUROSCI.0620-21.2021 PMC8612473

[60] Hevner RF, Haydar TF. The (not necessarily) convoluted role of basal radial glia in cortical neurogenesis. Cereb Cortex. 2012 Feb;22(2):465–8. 22116731 10.1093/cercor/bhr336 PMC3256413

[61] Florio M, Huttner WB. Neural progenitors, neurogenesis and the evolution of the neocortex. Development. 2014 Jun;141(11):2182–94. 24866113 10.1242/dev.090571

[62] Karpinski BA, Bryan CA, Paronett EM, Baker JL, Fernandez A, Horvath A, A cellular and molecular mosaic establishes growth and differentiation states for cranial sensory neurons. Dev Biol. 2016 Jul 15;415(2):228–41. 26988119 10.1016/j.ydbio.2016.03.015 PMC5091808

[63] Frank DU, Fotheringham LK, Brewer JA, Muglia LJ, Tristani-Firouzi M, Capecchi MR, An Fgf8 mouse mutant phenocopies human 22q11 deletion syndrome. Development. 2002 Oct;129(19):4591–603. 12223415 10.1242/dev.129.19.4591 PMC1876665

[64] Maynard TM, Gopalakrishna D, Meechan DW, Paronett EM, Newbern JM, LaMantia AS. 22q11 Gene dosage establishes an adaptive range for sonic hedgehog and retinoic acid signaling during early development. Hum Mol Genet. 2013 Jan 15;22(2):300–12. 23077214 10.1093/hmg/dds429 PMC3526161

[65] Zhang Y, Liu G, Guo T, Liang XG, Du H, Yang L, Cortical neural stem cell lineage progression is regulated by extrinsic signaling molecule sonic hedgehog. Cell Rep. 2020 Mar 31;30(13):4490–504.e4. 32234482 10.1016/j.celrep.2020.03.027 PMC7197103

[66] Vitelli F, Taddei I, Morishima M, Meyers EN, Lindsay EA, Baldini A. A genetic link between Tbx1 and fibroblast growth factor signaling. Development. 2002 Oct;129(19):4605–11. 12223416 10.1242/dev.129.19.4605

[67] Vermot J, Niederreither K, Garnier JM, Chambon P, Dolle P. Decreased embryonic retinoic acid synthesis results in a DiGeorge syndrome phenotype in newborn mice. Proc Natl Acad Sci U S A. 2003 Feb 18;100(4):1763–8. 12563036 10.1073/pnas.0437920100 PMC149907

[68] Bachiller D, Klingensmith J, Shneyder N, Tran U, Anderson R, Rossant J, The role of chordin/Bmp signals in mammalian pharyngeal development and DiGeorge syndrome. Development. 2003 Aug;130(15):3567–78. 12810603 10.1242/dev.00581

[69] Guris DL, Duester G, Papaioannou VE, Imamoto A. Dose-dependent interaction of Tbx1 and Crkl and locally aberrant RA signaling in a model of del22q11 syndrome. Dev Cell. 2006 Jan;10(1):81–92. 16399080 10.1016/j.devcel.2005.12.002

[70] Mehler MF, Rozental R, Dougherty M, Spray DC, Kessler JA. Cytokine regulation of neuronal differentiation of hippocampal progenitor cells. Nature. 1993 Mar 4;362(6415):62–5. 8383296 10.1038/362062a0

[71] Mehler MF, Kessler JA. Hematolymphopoietic and inflammatory cytokines in neural development. Trends Neurosci. 1997 Aug;20(8):357–65. 9246730 10.1016/s0166-2236(96)01045-4

[72] Qian X, Shen Q, Goderie SK, He W, Capela A, Davis AA, Timing of CNS cell generation: a programmed sequence of neuron and glial cell production from isolated murine cortical stem cells. Neuron. 2000 Oct;28(1):69–80. 11086984 10.1016/s0896-6273(00)00086-6

[73] Le Bras B, Barallobre MJ, Homman-Ludiye J, Ny A, Wyns S, Tammela T, VEGF-C is a trophic factor for neural progenitors in the vertebrate embryonic brain. Nat Neurosci. 2006 Mar;9(3):340–8. 16462734 10.1038/nn1646

[74] LaMantia AS. The usual suspects: GABA and glutamate may regulate proliferation in the neocortex. Neuron. 1995 Dec;15(6):1223–5. 8845147 10.1016/0896-6273(95)90002-0

[75] Meechan DW, Maynard TM, Tucker ES, Fernandez A, Karpinski BA, Rothblat LA, Modeling a model: mouse genetics, 22q11.2 Deletion Syndrome, and disorders of cortical circuit development. Prog Neurobiol. 2015 Jul;130:1–28. 25866365 10.1016/j.pneurobio.2015.03.004 PMC5019355

[76] Bhasin N, Maynard TM, Gallagher PA, LaMantia AS. Mesenchymal/epithelial regulation of retinoic acid signaling in the olfactory placode. Dev Biol. 2003 Sep 1;261(1):82–98. 12941622 10.1016/s0012-1606(03)00295-1

[77] Mackenzie F, Ruhrberg C. Diverse roles for VEGF-A in the nervous system. Development. 2012 Apr;139(8):1371–80. 22434866 10.1242/dev.072348

[78] Murphy M, Dutton R, Koblar S, Cheema S, Bartlett P. Cytokines which signal through the LIF receptor and their actions in the nervous system. Prog Neurobiol. 1997 Aug;52(5):355–78. 9304697 10.1016/s0301-0082(97)00020-8

[79] Moody SA, LaMantia AS. Transcriptional regulation of cranial sensory placode development. Curr Top Dev Biol. 2015;111:301–50. 25662264 10.1016/bs.ctdb.2014.11.009 PMC4425424

[80] Chau KF, Springel MW, Broadbelt KG, Park HY, Topal S, Lun MP, Progressive differentiation and instructive capacities of amniotic fluid and cerebrospinal fluid proteomes following neural tube closure. Dev Cell. 2015 Dec 21;35(6):789–802. 26702835 10.1016/j.devcel.2015.11.015 PMC4691285

[81] Lehtinen MK, Zappaterra MW, Chen X, Yang YJ, Hill AD, Lun M, The cerebrospinal fluid provides a proliferative niche for neural progenitor cells. Neuron. 2011 Mar 10;69(5):893–905. 21382550 10.1016/j.neuron.2011.01.023 PMC3085909

[82] Wechsler-Reya RJ, Scott MP. Control of neuronal precursor proliferation in the cerebellum by Sonic Hedgehog. Neuron. 1999 Jan;22(1):103–14. 10027293 10.1016/s0896-6273(00)80682-0

[83] Wallace VA. Purkinje-cell-derived Sonic hedgehog regulates granule neuron precursor cell proliferation in the developing mouse cerebellum. Curr Biol. 1999 Apr 22;9(8):445–8. 10226030 10.1016/s0960-9822(99)80195-x

[84] Henion PD, Weston JA. Retinoic acid selectively promotes the survival and proliferation of neurogenic precursors in cultured neural crest cell populations. Dev Biol. 1994 Jan;161(1):243–50. 8293876 10.1006/dbio.1994.1024

[85] Melton KR, Iulianella A, Trainor PA. Gene expression and regulation of hindbrain and spinal cord development. Front Biosci. 2004 Jan 1;9:117–38. 14766352 10.2741/1202

[86] Morimoto K, Nakajima K. Role of the immune system in the development of the central nervous system. Front Neurosci. 2019;13:916. 31551681 10.3389/fnins.2019.00916 PMC6735264

[87] Paredes I, Himmels P, Ruiz de Almodovar C. Neurovascular communication during CNS development. Dev Cell. 2018 Apr 9;45(1):10–32. 29634931 10.1016/j.devcel.2018.01.023

[88] Vogenstahl J, Parrilla M, Acker-Palmer A, Segarra M. Vascular regulation of developmental neurogenesis. Front Cell Dev Biol. 2022;10:890852. 35573692 10.3389/fcell.2022.890852 PMC9099230

[89] Conover JC, Doetsch F, Garcia-Verdugo JM, Gale NW, Yancopoulos GD, Alvarez-Buylla A. Disruption of Eph/ephrin signaling affects migration and proliferation in the adult subventricular zone. Nat Neurosci. 2000 Nov;3(11):1091–7. 11036265 10.1038/80606

[90] Klein R. Eph/ephrin signaling in morphogenesis, neural development and plasticity. Curr Opin Cell Biol. 2004 Oct;16(5):580–9. 15363810 10.1016/j.ceb.2004.07.002

[91] Woodhead GJ, Mutch CA, Olson EC, Chenn A. Cell-autonomous beta-catenin signaling regulates cortical precursor proliferation. J Neurosci. 2006 Nov 29;26(48):12620–30. 17135424 10.1523/JNEUROSCI.3180-06.2006 PMC2867669

[92] Mione MC, Cavanagh JF, Harris B, Parnavelas JG. Cell fate specification and symmetrical/asymmetrical divisions in the developing cerebral cortex. J Neurosci. 1997 Mar 15;17(6):2018–29. 9045730 10.1523/JNEUROSCI.17-06-02018.1997 PMC6793772

[93] Noctor SC, Martinez-Cerdeno V, Ivic L, Kriegstein AR. Cortical neurons arise in symmetric and asymmetric division zones and migrate through specific phases. Nat Neurosci. 2004 Feb;7(2):136–44. 14703572 10.1038/nn1172

[94] Huttner WB, Kosodo Y. Symmetric versus asymmetric cell division during neurogenesis in the developing vertebrate central nervous system. Curr Opin Cell Biol. 2005 Dec;17(6):648–57. 16243506 10.1016/j.ceb.2005.10.005

[95] Englund C, Fink A, Lau C, Pham D, Daza RA, Bulfone A, Pax6, Tbr2, and Tbr1 are expressed sequentially by radial glia, intermediate progenitor cells, and postmitotic neurons in developing neocortex. J Neurosci. 2005 Jan 5;25(1):247–51. 15634788 10.1523/JNEUROSCI.2899-04.2005 PMC6725189

[96] Noctor SC, Flint AC, Weissman TA, Wong WS, Clinton BK, Kriegstein AR. Dividing precursor cells of the embryonic cortical ventricular zone have morphological and molecular characteristics of radial glia. J Neurosci. 2002 Apr 15;22(8):3161–73. 11943818 10.1523/JNEUROSCI.22-08-03161.2002 PMC6757532

[97] Bertrand N, Castro DS, Guillemot F. Proneural genes and the specification of neural cell types. Nat Rev Neurosci. 2002 Jul;3(7):517–30. 12094208 10.1038/nrn874

[98] Guillamon-Vivancos T, Tyler WA, Medalla M, Chang WW, Okamoto M, Haydar TF, Distinct neocortical progenitor lineages fine-tune neuronal diversity in a layer-specific manner. Cereb Cortex. 2019 Mar 1;29(3):1121–38. 29415216 10.1093/cercor/bhy019 PMC6373699

[99] Kriegstein A, Alvarez-Buylla A. The glial nature of embryonic and adult neural stem cells. Annu Rev Neurosci. 2009;32:149–84. 19555289 10.1146/annurev.neuro.051508.135600 PMC3086722

[100] Kempermann G, Song H, Gage FH. Neurogenesis in the adult Hippocampus. Cold Spring Harb Perspect Biol. 2015 Sep 1;7(9):a018812. 26330519 10.1101/cshperspect.a018812 PMC4563705

[101] Obernier K, Alvarez-Buylla A. Neural stem cells: origin, heterogeneity and regulation in the adult mammalian brain. Development. 2019 Feb 18;146(4):dev156059. 30777863 10.1242/dev.156059 PMC6398449

[102] Furlan A, Adameyko I. Schwann cell precursor: a neural crest cell in disguise? Dev Biol. 2018 Dec 1;444(Suppl 1):S25–35. 29454705 10.1016/j.ydbio.2018.02.008

[103] Schwob JE, Jang W, Holbrook EH, Lin B, Herrick DB, Peterson JN, Stem and progenitor cells of the mammalian olfactory epithelium: taking poietic license. J Comp Neurol. 2017 Mar 1;525(4):1034–54. 27560601 10.1002/cne.24105 PMC5805156

[104] Anderson DJ. The neural crest cell lineage problem: neuropoiesis? Neuron. 1989 Jul;3(1):1–12. 2695146 10.1016/0896-6273(89)90110-4

[105] Scheffler B, Horn M, Blumcke I, Laywell ED, Coomes D, Kukekov VG, Marrow-mindedness: a perspective on neuropoiesis. Trends Neurosci. 1999 Aug;22(8):348–57. 10407420 10.1016/s0166-2236(99)01416-2

[106] Terskikh AV, Easterday MC, Li L, Hood L, Kornblum HI, Geschwind DH, From hematopoiesis to neuropoiesis: evidence of overlapping genetic programs. Proc Natl Acad Sci U S A. 2001 Jul 3;98(14):7934–9. 11438738 10.1073/pnas.131200898 PMC35446

[107] Scheffler B, Walton NM, Lin DD, Goetz AK, Enikolopov G, Roper SN, Phenotypic and functional characterization of adult brain neuropoiesis. Proc Natl Acad Sci U S A. 2005 Jun 28;102(26):9353–8. 15961540 10.1073/pnas.0503965102 PMC1150897

[108] Liggett LA, Sankaran VG. Unraveling hematopoiesis through the lens of genomics. Cell. 2020 Sep 17;182(6):1384–400. 32946781 10.1016/j.cell.2020.08.030 PMC7508400

[109] Sir JH, Barr AR, Nicholas AK, Carvalho OP, Khurshid M, Sossick A, A primary microcephaly protein complex forms a ring around parental centrioles. Nat Genet. 2011 Oct 9;43(11):1147–53. 21983783 10.1038/ng.971 PMC3299569

[110] Yang YJ, Baltus AE, Mathew RS, Murphy EA, Evrony GD, Gonzalez DM, Microcephaly gene links trithorax and REST/NRSF to control neural stem cell proliferation and differentiation. Cell. 2012 Nov 21;151(5):1097–112. 23178126 10.1016/j.cell.2012.10.043 PMC3567437

[111] Marthiens V, Rujano MA, Pennetier C, Tessier S, Paul-Gilloteaux P, Basto R. Centrosome amplification causes microcephaly. Nat Cell Biol. 2013 Jul;15(7):731–40. 23666084 10.1038/ncb2746

[112] Fregeac J, Moriceau S, Poli A, Nguyen LS, Oury F, Colleaux L. Loss of the neurodevelopmental disease-associated gene miR-146a impairs neural progenitor differentiation and causes learning and memory deficits. Mol Autism. 2020 Mar 30;11(1):22. 32228681 10.1186/s13229-020-00328-3 PMC7106595

[113] Parenti I, Rabaneda LG, Schoen H, Novarino G. Neurodevelopmental disorders: from genetics to functional pathways. Trends Neurosci. 2020 Aug;43(8):608–21. 32507511 10.1016/j.tins.2020.05.004

[114] Gandal MJ, Geschwind DH. Polygenicity in psychiatry-like it or not, we have to understand it. Biol Psychiatry. 2021 Jan 1;89(1):2–4. 33272361 10.1016/j.biopsych.2020.10.002

[115] Poelmans G, Pauls DL, Buitelaar JK, Franke B. Integrated genome-wide association study findings: identification of a neurodevelopmental network for attention deficit hyperactivity disorder. Am J Psychiatry. 2011 Apr;168(4):365–77. 21324949 10.1176/appi.ajp.2010.10070948

[116] Thapar A, Martin J, Mick E, Arias Vasquez A, Langley K, Scherer SW, Psychiatric gene discoveries shape evidence on ADHD’s biology. Mol Psychiatry. 2016 Sep;21(9):1202–7. 26573769 10.1038/mp.2015.163 PMC4820035

[117] Geschwind DH. Genetics of autism spectrum disorders. Trends Cogn Sci. 2011 Sep;15(9):409–16. 21855394 10.1016/j.tics.2011.07.003 PMC3691066

[118] Gandal MJ, Haney JR, Parikshak NN, Leppa V, Ramaswami G, Hartl C, Shared molecular neuropathology across major psychiatric disorders parallels polygenic overlap. Science. 2018 Feb 9;359(6376):693–7. 29439242 10.1126/science.aad6469 PMC5898828

[119] Gandal MJ, Zhang P, Hadjimichael E, Walker RL, Chen C, Liu S, Transcriptome-wide isoform-level dysregulation in ASD, schizophrenia, and bipolar disorder. Science. 2018 Dec 14;362(6420):eaat8127. 30545856 10.1126/science.aat8127 PMC6443102

[120] Carlson C, Sirotkin H, Pandita R, Goldberg R, McKie J, Wadey R, Molecular definition of 22q11 deletions in 151 velo-cardio-facial syndrome patients. Am J Hum Genet. 1997 Sep;61(3):620–9. 9326327 10.1086/515508 PMC1715959

[121] Maynard TM, Haskell GT, Bhasin N, Lee JM, Gassman AA, Lieberman JA, RanBP1, a velocardiofacial/DiGeorge syndrome candidate gene, is expressed at sites of mesenchymal/epithelial induction. Mech Dev. 2002 Feb;111(1–2):177–80. 11804793 10.1016/s0925-4773(01)00616-5

[122] Karpinski BA, Maynard TM, Fralish MS, Nuwayhid S, Zohn IE, Moody SA, Dysphagia and disrupted cranial nerve development in a mouse model of DiGeorge (22q11) deletion syndrome. Dis Model Mech. 2014 Feb;7(2):245–57. 24357327 10.1242/dmm.012484 PMC3917245

[123] Maynard TM, Horvath A, P Bernot J, Karpinski BA, Tavares ALP, Shah A, Transcriptional dysregulation in developing trigeminal sensory neurons in the LgDel mouse model of DiGeorge 22q11.2 deletion syndrome. Hum Mol Genet. 2020 Apr 15;29(6):1002–17. 32047912 10.1093/hmg/ddaa024 PMC7158380

[124] Motahari Z, Moody SA, Maynard TM, LaMantia AS. In the line-up: deleted genes associated with DiGeorge/22q11.2 deletion syndrome: are they all suspects? J Neurodev Disord. 2019 Jun 7;11(1):7. 31174463 10.1186/s11689-019-9267-z PMC6554986

[125] Visel A, Thaller C, Eichele G. GenePaint.org: an atlas of gene expression patterns in the mouse embryo. Nucleic Acids Res. 2004 Jan 1;32(Database issue):D552–6. (Database issue). 14681479 10.1093/nar/gkh029 PMC308763

[126] Maynard TM, Zohn IE, Moody SA, LaMantia AS. Suckling, feeding, and swallowing: behaviors, circuits, and targets for neurodevelopmental pathology. Annu Rev Neurosci. 2020 Jul 8;43:315–36. 32101484 10.1146/annurev-neuro-100419-100636 PMC7359496

[127] Morrow B, Goldberg R, Carlson C, Das Gupta R, Sirotkin H, Collins J, Molecular definition of the 22q11 deletions in velo-cardio-facial syndrome. Am J Hum Genet. 1995 Jun;56(6):1391–403. 7762562 PMC1801093

[128] Parsons JT, Wilkerson V, Parsons SJ. Structural and functional motifs of the Rous sarcoma virus src protein. Gene Amplif Anal. 1986;4:1–19. 2851527

[129] Broderick R, Nasheuer HP. Regulation of Cdc45 in the cell cycle and after DNA damage. Biochem Soc Trans. 2009 Aug;37(Pt 4):926–30. 19614620 10.1042/BST0370926

[130] Chang YH, Nishimura S, Oishi H, Kelly VP, Kuno A, Takahashi S. TRMT2A is a novel cell cycle regulator that suppresses cell proliferation. Biochem Biophys Res Commun. 2019 Jan 8;508(2):410–5. 30502085 10.1016/j.bbrc.2018.11.104

[131] Maynard TM, Meechan DW, Dudevoir ML, Gopalakrishna D, Peters AZ, Heindel CC, Mitochondrial localization and function of a subset of 22q11 deletion syndrome candidate genes. Mol Cell Neurosci. 2008 Nov;39(3):439–51. 18775783 10.1016/j.mcn.2008.07.027 PMC2729512

[132] Favicchia I, Flore G, Cioffi S, Lania G, Baldini A, Illingworth E. Pharmacological rescue of the brain cortex phenotype of Tbx1 mouse mutants: significance for 22q11.2 deletion syndrome. Front Mol Neurosci. 2021;14:663598. 34552467 10.3389/fnmol.2021.663598 PMC8450345

[133] Flore G, Cioffi S, Bilio M, Illingworth E. Cortical development requires mesodermal expression of Tbx1, a gene haploinsufficient in 22q11.2 deletion syndrome. Cereb Cortex. 2017 Mar 1;27(3):2210–25. 27005988 10.1093/cercor/bhw076

[134] Molnar Z, Clowry G. Cerebral cortical development in rodents and primates. Prog Brain Res. 2012;195:45–70. 22230622 10.1016/B978-0-444-53860-4.00003-9

[135] Pierri JN, Volk CL, Auh S, Sampson A, Lewis DA. Decreased somal size of deep layer 3 pyramidal neurons in the prefrontal cortex of subjects with schizophrenia. Arch Gen Psychiatry. 2001 May;58(5):466–73. 11343526 10.1001/archpsyc.58.5.466

[136] Sohal VS, Rubenstein JLR. Excitation-inhibition balance as a framework for investigating mechanisms in neuropsychiatric disorders. Mol Psychiatry. 2019 Sep;24(9):1248–57. 31089192 10.1038/s41380-019-0426-0 PMC6742424

[137] Brigman JL, Graybeal C, Holmes A. Predictably irrational: assaying cognitive inflexibility in mouse models of schizophrenia. Front Neurosci. 2010;4:13. 20859447 10.3389/neuro.01.013.2010 PMC2938983

[138] Bussey TJ, Saksida LM, Rothblat LA. Discrimination of computer-graphic stimuli by mice: a method for the behavioral characterization of transgenic and gene-knockout models. Behav Neurosci. 2001 Aug;115(4):957–60. 11508736 10.1037//0735-7044.115.4.957

[139] Meechan DW, Tucker ES, Maynard TM, LaMantia AS. Cxcr4 regulation of interneuron migration is disrupted in 22q11.2 deletion syndrome. Proc Natl Acad Sci U S A. 2012 Nov 6;109(45):18601–6. 23091025 10.1073/pnas.1211507109 PMC3494945

[140] Meechan DW, Rutz HL, Fralish MS, Maynard TM, Rothblat LA, LaMantia AS. Cognitive ability is associated with altered medial frontal cortical circuits in the LgDel mouse model of 22q11.2DS. Cereb Cortex. 2015 May;25(5):1143–51. 24217989 10.1093/cercor/bht308 PMC4397569

[141] Niesler B, Rappold GA. Emerging evidence for gene mutations driving both brain and gut dysfunction in autism spectrum disorder. Mol Psychiatry. 2021 May;26(5):1442–4. 32461615 10.1038/s41380-020-0778-5 PMC8159735

[142] McChesney N, Barth JL, Rumschlag JA, Tan J, Harrington AJ, Noble KV, Peripheral auditory nerve impairment in a mouse model of syndromic autism. J Neurosci. 2022 Oct 19;42(42):8002–18. 36180228 10.1523/JNEUROSCI.0253-22.2022 PMC9617620

[143] Welby L, Caudill H, Yitsege G, Hamad A, Bunyak F, Zohn IE, Persistent feeding and swallowing deficits in a mouse model of 22q11.2 deletion syndrome. Front Neurol. 2020;11:4. 32082240 10.3389/fneur.2020.00004 PMC7006055

[144] Ayer-Le Lievre CS, Le Douarin NM. The early development of cranial sensory ganglia and the potentialities of their component cells studied in quail-chick chimeras. Dev Biol. 1982 Dec;94(2):291–310. 7152108 10.1016/0012-1606(82)90349-9

[145] D’Amico-Martel A, Noden DM. Contributions of placodal and neural crest cells to avian cranial peripheral ganglia. Am J Anat. 1983 Apr;166(4):445–68. 6858941 10.1002/aja.1001660406

[146] Motahari Z, Maynard TM, Popratiloff A, Moody SA, LaMantia AS. Aberrant early growth of individual trigeminal sensory and motor axons in a series of mouse genetic models of 22q11.2 deletion syndrome. Hum Mol Genet. 2020 Nov 4;29(18):3081–93. 32901287 10.1093/hmg/ddaa199 PMC7645708

[147] Yitsege G, Stokes BA, Sabatino JA, Sugrue KF, Banyai G, Paronett EM, Variations in maternal vitamin A intake modifies phenotypes in a mouse model of 22q11.2 deletion syndrome. Birth Defects Res. 2020 Oct;112(16):1194–208. 32431076 10.1002/bdr2.1709 PMC7586978

[148] Yoshida K, Kuo F, George EL, Sharpe AH, Dutta A. Requirement of CDC45 for postimplantation mouse development. Mol Cell Biol. 2001 Jul;21(14):4598–603. 11416137 10.1128/MCB.21.14.4598-4603.2001 PMC87121

[149] Tedeschi A, Ciciarello M, Mangiacasale R, Roscioli E, Rensen WM, Lavia P. RANBP1 localizes a subset of mitotic regulatory factors on spindle microtubules and regulates chromosome segregation in human cells. J Cell Sci. 2007 Nov 1;120(Pt 21):3748–61. 17940066 10.1242/jcs.009308

[150] Fernandez A, Meechan DW, Karpinski BA, Paronett EM, Bryan CA, Rutz HL, Mitochondrial dysfunction leads to cortical under-connectivity and cognitive impairment. Neuron. 2019 Jun 19;102(6):1127–42.e3. 31079872 10.1016/j.neuron.2019.04.013 PMC6668992

[151] Pandur PD, Moody SA. Xenopus Six1 gene is expressed in neurogenic cranial placodes and maintained in the differentiating lateral lines. Mech Dev. 2000 Sep;96(2):253–7. 10960794 10.1016/s0925-4773(00)00396-8

[152] Schlosser G, Awtry T, Brugmann SA, Jensen ED, Neilson K, Ruan G, Eya1 and Six1 promote neurogenesis in the cranial placodes in a SoxB1-dependent fashion. Dev Biol. 2008 Aug 1;320(1):199–214. 18571637 10.1016/j.ydbio.2008.05.523 PMC2671077

[153] Gotz M, Stoykova A, Gruss P. Pax6 controls radial glia differentiation in the cerebral cortex. Neuron. 1998 Nov;21(5):1031–44. 9856459 10.1016/s0896-6273(00)80621-2

[154] Goulding MD, Lumsden A, Gruss P. Signals from the notochord and floor plate regulate the region-specific expression of two Pax genes in the developing spinal cord. Development. 1993 Mar;117(3):1001–16. 8100762 10.1242/dev.117.3.1001

[155] Guillemot F, Joyner AL. Dynamic expression of the murine Achaete-Scute homologue Mash-1 in the developing nervous system. Mech Dev. 1993 Aug;42(3):171–85. 8217843 10.1016/0925-4773(93)90006-j

[156] Lee JE, Hollenberg SM, Snider L, Turner DL, Lipnick N, Weintraub H. Conversion of Xenopus ectoderm into neurons by NeuroD, a basic helix-loop-helix protein. Science. 1995 May 12;268(5212):836–44. 7754368 10.1126/science.7754368

[157] Shen Q, Wang Y, Dimos JT, Fasano CA, Phoenix TN, Lemischka IR, The timing of cortical neurogenesis is encoded within lineages of individual progenitor cells. Nat Neurosci. 2006 Jun;9(6):743–51. 16680166 10.1038/nn1694

[158] Noctor SC, Flint AC, Weissman TA, Dammerman RS, Kriegstein AR. Neurons derived from radial glial cells establish radial units in neocortex. Nature. 2001 Feb 8;409(6821):714–20. 11217860 10.1038/35055553

[159] Shine HD, Sidman RL. Immunoreactive myelin basic proteins are not detected when shiverer mutant Schwann cells and fibroblasts are co-cultured with normal neurons. J Cell Biol. 1984 Apr;98(4):1291–5. 6201490 10.1083/jcb.98.4.1291 PMC2113205

[160] Penisson M, Jin M, Wang S, Hirotsune S, Francis F, Belvindrah R. Lis1 mutation prevents basal radial glia-like cell production in the mouse. Hum Mol Genet. 2022 Mar 21;31(6):942–57. 34635911 10.1093/hmg/ddab295

[161] Yao H, Hannum DF, Zhai Y, Hill SF, Albanus RD, Lou W, CHD7 promotes neural progenitor differentiation in embryonic stem cells via altered chromatin accessibility and nascent gene expression. Sci Rep. 2020 Oct 15;10(1):17445. 33060836 10.1038/s41598-020-74537-4 PMC7562747

[162] Geng A, Qiu R, Murai K, Liu J, Wu X, Zhang H, KIF20A/MKLP2 regulates the division modes of neural progenitor cells during cortical development. Nat Commun. 2018 Jul 13;9(1):2707. 30006548 10.1038/s41467-018-05152-1 PMC6045631

[163] Nishino J, Saunders TL, Sagane K, Morrison SJ. Lgi4 promotes the proliferation and differentiation of glial lineage cells throughout the developing peripheral nervous system. J Neurosci. 2010 Nov 10;30(45):15228–40. 21068328 10.1523/JNEUROSCI.2286-10.2010 PMC3059102

[164] Williams JP, Wu J, Johansson G, Rizvi TA, Miller SC, Geiger H, Nf1 mutation expands an EGFR-dependent peripheral nerve progenitor that confers neurofibroma tumorigenic potential. Cell Stem Cell. 2008 Dec 4;3(6):658–69. 19041782 10.1016/j.stem.2008.10.003 PMC3487385

[165] Da Silva F, Zhang K, Pinson A, Fatti E, Wilsch-Brauninger M, Herbst J, Mitotic WNT signalling orchestrates neurogenesis in the developing neocortex. EMBO J. 2021 Oct 1;40(19):e108041. 34431536 10.15252/embj.2021108041 PMC8488556

[166] Komada M, Saitsu H, Kinboshi M, Miura T, Shiota K, Ishibashi M. Hedgehog signaling is involved in development of the neocortex. Development. 2008 Aug;135(16):2717–27. 18614579 10.1242/dev.015891

[167] Zhang C, Tu HL, Jia G, Mukhtar T, Taylor V, Rzhetsky A, Ultra-multiplexed analysis of single-cell dynamics reveals logic rules in differentiation. Sci Adv. 2019 Apr;5(4):eaav7959. 30949582 10.1126/sciadv.aav7959 PMC6447378

[168] Ruan X, Kang B, Qi C, Lin W, Wang J, Zhang X. Progenitor cell diversity in the developing mouse neocortex. Proc Natl Acad Sci U S A. 2021 Mar 9;118(10):e2018866118. 33649223 10.1073/pnas.2018866118 PMC7958455

[169] Li Z, Tyler WA, Zeldich E, Santpere Baro G, Okamoto M, Gao T, Transcriptional priming as a conserved mechanism of lineage diversification in the developing mouse and human neocortex. Sci Adv. 2020 Nov;6(45):eabd2068. 33158872 10.1126/sciadv.abd2068 PMC7673705

[170] Mukhtar T, Breda J, Adam MA, Boareto M, Grobecker P, Karimaddini Z, Temporal and sequential transcriptional dynamics define lineage shifts in corticogenesis. EMBO J. 2022 Dec 15;41(24):e111132. 36345783 10.15252/embj.2022111132 PMC9753470

[171] Kerimoglu C, Pham L, Tonchev AB, Sakib MS, Xie Y, Sokpor G, H3 acetylation selectively promotes basal progenitor proliferation and neocortex expansion. Sci Adv. 2021 Sep 17;7(38):eabc6792. 34524839 10.1126/sciadv.abc6792 PMC8443185

[172] Zhong S, Zhang S, Fan X, Wu Q, Yan L, Dong J, A single-cell RNA-seq survey of the developmental landscape of the human prefrontal cortex. Nature. 2018 Mar 22;555(7697):524–8. 29539641 10.1038/nature25980

[173] Johnson MB, Wang PP, Atabay KD, Murphy EA, Doan RN, Hecht JL, Single-cell analysis reveals transcriptional heterogeneity of neural progenitors in human cortex. Nat Neurosci. 2015 May;18(5):637–46. 25734491 10.1038/nn.3980 PMC5568903

[174] Loo L, Simon JM, Xing L, McCoy ES, Niehaus JK, Guo J, Single-cell transcriptomic analysis of mouse neocortical development. Nat Commun. 2019 Jan 11;10(1):134. 30635555 10.1038/s41467-018-08079-9 PMC6329831

[175] Shibata M, Nakao H, Kiyonari H, Abe T, Aizawa S. MicroRNA-9 regulates neurogenesis in mouse telencephalon by targeting multiple transcription factors. J Neurosci. 2011 Mar 2;31(9):3407–22. 21368052 10.1523/JNEUROSCI.5085-10.2011 PMC6623912

[176] Miller BH, Zeier Z, Xi L, Lanz TA, Deng S, Strathmann J, MicroRNA-132 dysregulation in schizophrenia has implications for both neurodevelopment and adult brain function. Proc Natl Acad Sci U S A. 2012 Feb 21;109(8):3125–30. 22315408 10.1073/pnas.1113793109 PMC3286960

[177] Lennox AL, Mao H, Silver DL. RNA on the brain: emerging layers of post-transcriptional regulation in cerebral cortex development. WIREs Dev Biol. 2018 Jan;7(1):e290. 10.1002/wdev.290 PMC5746464 28837264

[178] Pereira JD, Sansom SN, Smith J, Dobenecker MW, Tarakhovsky A, Livesey FJ. Ezh2, the histone methyltransferase of PRC2, regulates the balance between self-renewal and differentiation in the cerebral cortex. Proc Natl Acad Sci U S A. 2010 Sep 7;107(36):15957–62. 20798045 10.1073/pnas.1002530107 PMC2936600

[179] Amberg N, Pauler FM, Streicher C, Hippenmeyer S. Tissue-wide genetic and cellular landscape shapes the execution of sequential PRC2 functions in neural stem cell lineage progression. Sci Adv. 2022 Nov 4;8(44):eabq1263. 36322669 10.1126/sciadv.abq1263 PMC9629739

[180] Albert M, Kalebic N, Florio M, Lakshmanaperumal N, Haffner C, Brandl H, Epigenome profiling and editing of neocortical progenitor cells during development. EMBO J. 2017 Sep 1;36(17):2642–58. 28765163 10.15252/embj.201796764 PMC5579386

[181] Albert M, Huttner WB. Epigenetic and transcriptional pre-patterning-an emerging theme in cortical neurogenesis. Front Neurosci. 2018;12:359. 29896084 10.3389/fnins.2018.00359 PMC5986960

[182] Sanosaka T, Imamura T, Hamazaki N, Chai M, Igarashi K, Ideta-Otsuka M, DNA methylome analysis identifies transcription factor-based epigenomic signatures of multilineage competence in neural stem/progenitor cells. Cell Rep. 2017 Sep 19;20(12):2992–3003. 28930691 10.1016/j.celrep.2017.08.086

[183] Hahn MA, Jin SG, Li AX, Liu J, Huang Z, Wu X, Reprogramming of DNA methylation at NEUROD2-bound sequences during cortical neuron differentiation. Sci Adv. 2019 Oct;5(10):eaax0080. 31681843 10.1126/sciadv.aax0080 PMC6810389

[184] Cook DL, Gerber AN, Tapscott SJ. Modeling stochastic gene expression: implications for haploinsufficiency. Proc Natl Acad Sci U S A. 1998 Dec 22;95(26):15641–6. 9861023 10.1073/pnas.95.26.15641 PMC28097

[185] Sigurdsson T, Stark KL, Karayiorgou M, Gogos JA, Gordon JA. Impaired hippocampal-prefrontal synchrony in a genetic mouse model of schizophrenia. Nature. 2010 Apr 1;464(7289):763–7. 20360742 10.1038/nature08855 PMC2864584

[186] Fenelon K, Xu B, Lai CS, Mukai J, Markx S, Stark KL, The pattern of cortical dysfunction in a mouse model of a schizophrenia-related microdeletion. J Neurosci. 2013 Sep 11;33(37):14825–39. 24027283 10.1523/JNEUROSCI.1611-13.2013 PMC3771024

[187] Fenelon K, Mukai J, Xu B, Hsu PK, Drew LJ, Karayiorgou M, Deficiency of Dgcr8, a gene disrupted by the 22q11.2 microdeletion, results in altered short-term plasticity in the prefrontal cortex. Proc Natl Acad Sci U S A. 2011 Mar 15;108(11):4447–52. 21368174 10.1073/pnas.1101219108 PMC3060227

